# Roles of mTOR in thoracic aortopathy understood by complex intracellular signaling interactions

**DOI:** 10.1371/journal.pcbi.1009683

**Published:** 2021-12-13

**Authors:** Ana C. Estrada, Linda Irons, Bruno V. Rego, Guangxin Li, George Tellides, Jay D. Humphrey

**Affiliations:** 1 Department of Biomedical Engineering, Yale University; New Haven, Connecticut, United States of America; 2 Department of Surgery, Yale School of Medicine; New Haven, Connecticut, United States of America; 3 Vascular Biology and Therapeutics Program, Yale School of Medicine; New Haven, Connecticut, United States of America; University of Pittsburgh, UNITED STATES

## Abstract

Thoracic aortopathy–aneurysm, dissection, and rupture–is increasingly responsible for significant morbidity and mortality. Advances in medical genetics and imaging have improved diagnosis and thus enabled earlier prophylactic surgical intervention in many cases. There remains a pressing need, however, to understand better the underlying molecular and cellular mechanisms with the hope of finding robust pharmacotherapies. Diverse studies in patients and mouse models of aortopathy have revealed critical changes in multiple smooth muscle cell signaling pathways that associate with disease, yet integrating information across studies and models has remained challenging. We present a new quantitative network model that includes many of the key smooth muscle cell signaling pathways and validate the model using a detailed data set that focuses on hyperactivation of the mechanistic target of rapamycin (mTOR) pathway and its inhibition using rapamycin. We show that the model can be parameterized to capture the primary experimental findings both qualitatively and quantitatively. We further show that simulating a population of cells by varying receptor reaction weights leads to distinct proteomic clusters within the population, and that these clusters emerge due to a bistable switch driven by positive feedback in the PI3K/AKT/mTOR signaling pathway.

## Author summary

Cell signaling drives changes across scales, from altered transcription at the single-cell level to tissue-level growth and remodeling. Studying complex interactions within cell signaling pathways can lead to a better understanding of the progression of disease. In particular, we are interested in how vascular cells can change their phenotype in a way that exacerbates aortopathy, namely, the development of aneurysms, dissections, and rupture. In this study we built a novel cell signaling network model of a vascular smooth muscle cell using archival data and used it to capture the effects of a genetic knock-out and subsequent pharmacologic rescue. We then used the model to simulate populations of smooth muscle cells and found that small perturbations to the strength of signaling can lead to distinct clusters of cells. With further analysis of the network substructures, we found that a positive feedback loop within the network was responsible for the distinct phenotypes we saw in our clusters of simulated cells. We believe that this work not only helps us to understand changes in smooth muscle cell phenotype but also opens the possibility to study other signaling perturbations associated with aortopathy.

## Introduction

Smooth muscle cells (SMCs) of the arterial media serve as central nodes in vascular development, homeostasis, adaptation, and disease [1,2], acting in concert with endothelial cells of the intima and fibroblasts of the adventitia. Although all three cell types are involved in thoracic aortopathy–aneurysm, dissection, and rupture–it is widely held that SMC dysfunction plays a particularly critical role since early evidence of disease often presents as medial degeneration [3–5]. Under normal conditions in the healthy adult, aortic SMCs constantly assess their local micromechanical environment and either maintain or remodel the composition and structure of the extracellular matrix (ECM) so as to preserve both the geometry and key biomechanical properties [6], including the resilience and compliance that optimize the primary function of this conduit vessel. Dysfunctional SMCs are characterized by myriad changes in intracellular signaling, resulting in and from differentially expressed genes and associated altered gene products. Affected intracellular pathways in thoracic aortopathies include the mitogen activated protein kinases (MAPK), Smads, Rho/Rho kinase, and mechanistic target of rapamycin (mTOR), which together are responsible for the diverse changes in SMC processes that affect growth/proliferation, ECM deposition/degradation, actomyosin-based contractility, and cell survival [7–10].

Mouse models continue to provide important insight into underlying causes of both genetically triggered and induced thoracic aortopathies [11,12], yet in most cases attention has focused on alterations in one or two signaling pathways to render data interpretation tractable. Given the complex interactions across many pathways, there is a pressing need to synthesize findings and to understand disease progression from transcript to tissue. We suggest that such synthesis is now possible conceptually, namely, by melding information available from detailed biomechanical phenotyping of the vascular wall [13], in vivo imaging that enables detailed calculations of hemodynamics as a function of local wall properties [14], information on effects of matrix turnover on evolving vascular geometry and properties [15], and details on changes in cell signaling [16]. Fundamental to such a multiscale understanding is a detailed interpretation of interactions across the many relevant intracellular signaling pathways. Herein, we present a new SMC signaling model that is constructed based on findings in over 100 archival reports, then parameterized and validated using detailed data from a recent study that revealed a broader SMC phenotypic spectrum than previously appreciated [10]. Specifically, we include the multiple pathways noted above while focusing on mTOR.

The *Tor* genes were discovered in the early 1990s as targets of rapamycin, an antifungal metabolite produced by bacteria that was discovered in the 1970s on Easter Island (“Rapa Nui” in the native language) [17]. Briefly, the mTOR signaling pathway has long been appreciated as a central regulator of cell metabolism, growth/proliferation, and survival [17–19], though its biological impacts continue to expand, including SMC-mediated regulation of ECM within the medial layer of arteries [20,21]. Although mTOR signaling is complex, it is often conceptualized primarily in terms of the phospho-inositide-3-kinase (PI3K)/protein kinase B (AKT)/mechanistic target of rapamycin (mTOR) axis (S1 Fig), noting that mTOR presents as two protein complexes, raptor-associated mTOR complex 1 (mTORC1) and rictor-associated mTOR complex 2 (mTORC2). The tuberous sclerosis complex TSC1/2, consisting of hamartin, or TSC1, and tuberin, or TSC2, is a strong inhibitor of mTOR signaling. Hence, inactivation of TSC1/2 can hyperactivate mTOR signaling, especially evident via increased phosphorylation of S6 kinase (S6K) and eukaryote initiation factor 4E binding protein 1 (4EBP1), key downstream targets of mTORC1. Mouse models have revealed that mutations to *Tsc2* [22] and *Tsc1* [10] can lead to thoracic aortopathies, implicating a key role of mTOR signaling in promoting or preventing aortic wall integrity.

Of particular importance herein, Li and colleagues sought to understand better the role of vascular SMC proliferation on the development and progression of thoracic aortopathies by using a conditional knock-out (KO) of *Tsc1* in SMCs in male mice on a C57BL/6J background [10]. They induced the KO using tamoxifen, typically beginning at 1.5 weeks of age, and they measured resulting blood pressures as well as aortic morphology, composition, and cell signaling, among other metrics. As expected, KO of *Tsc1* hyperactivated mTORC1 in the aortic SMCs, demonstrated by increased levels of the downstream species p-S6K, p-S6, and p-4EBP1. These KO mice presented with aortic dissection, with incidence increasing with advancing age from approximately 25% at 12 weeks to 75% at 36 weeks of age. The age- and sex-matched control mice did not develop any aortopathy. In addition, the aortas of the KO mice showed significant dilation and reduced contractile capacity, the latter revealed by diminished levels of contractile proteins and reduced responses to vasoconstrictors. Even by 3 weeks of age, the KO mice had significantly lower expression of transcripts associated with matrix synthesis, specifically *Eln* and *Col3a1*. There was also significantly greater elastin fragmentation, perhaps exacerbated by the significantly higher expression of matrix metalloproteinase 2 (MMP2), and significantly more SMC proliferation and apoptosis. The degradative function of the KO SMCs was particularly striking. These cells expressed high levels of lysosome-related proteins, such as LAMP2 and MITF, had larger numbers of degradative organelles, and showed greater digestion of ECM components and erythrocytes. These behaviors indicate that this SMC-specific KO of *Tsc1* resulted in a phenotypic change distinct from traditional contractile-to-synthetic switching, resulting instead in an acquired degradative phenotype. Remarkably, the mTORC1-specific inhibitor rapamycin abolished many of these effects. We sought to capture the experimental changes seen in the KO SMCs using a new SMC signaling network model and then to explore the model parametrically to gain increased insight into this phenotype.

## Results

### The tuned network model qualitatively captures experimental changes seen in *Tsc1* KO aortas

**Fig 1:**
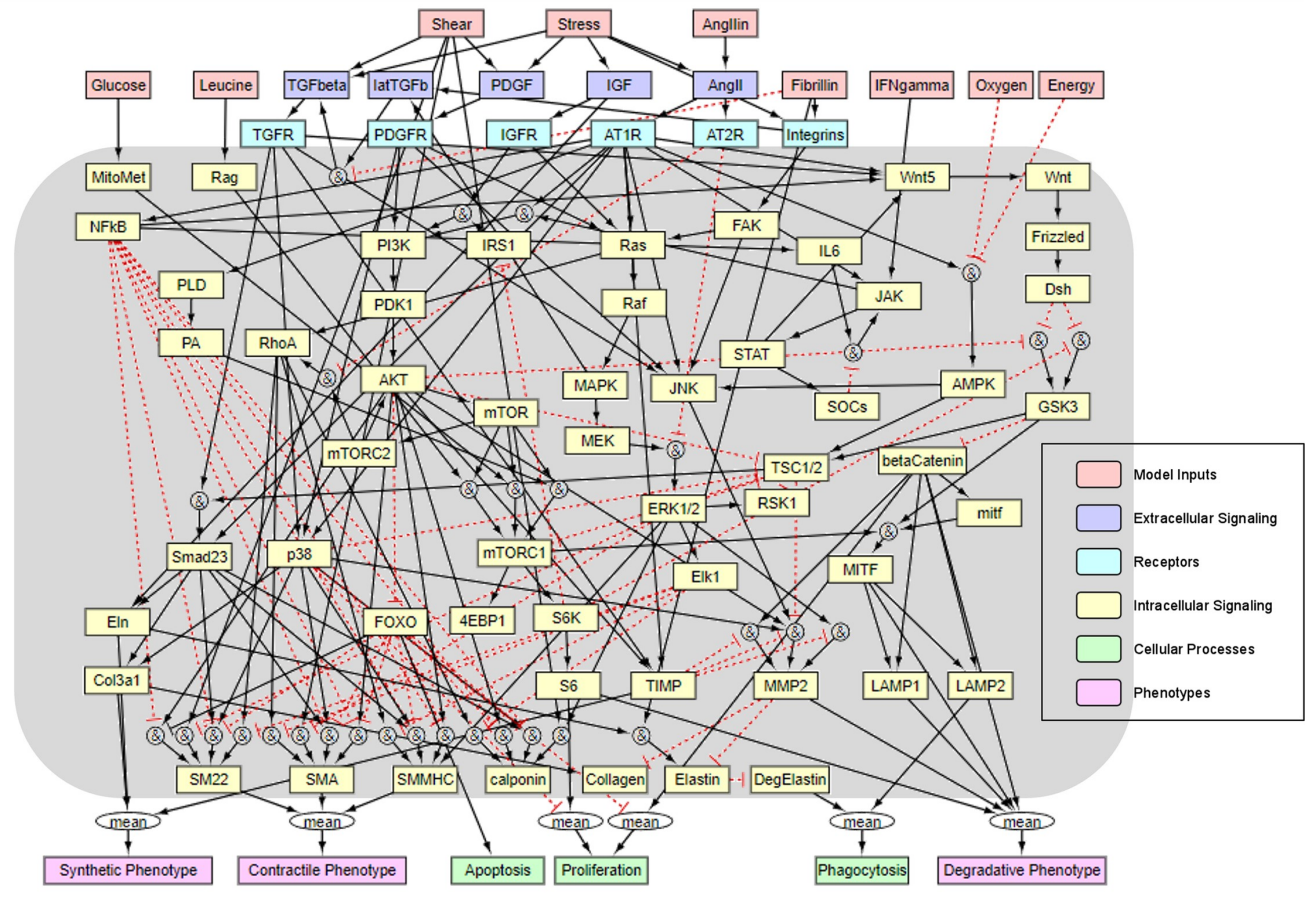
Schematic rendering of the smooth muscle cell network model featuring 81 nodes (species) and 138 edges (reactions), including model inputs (light red), extracellular signaling molecules (blue), cell-surface receptors (cyan), and intracellular signaling species (yellow). The activation level (bound from zero to one) of specific species was used to estimate the level of cellular processes (light green) and degree of phenotypic modulation (light purple) of the cell. See Table A in S1 Text for details on the reactions and the associated 105 references upon which this model was built.

Fig 1 shows the cell signaling model developed and used in this paper; associated model equations and parameters are in Methods, including Table 1. In addition to the PI3K/AKT/mTOR axis of primary interest (S1 Fig), other relevant signaling pathways (e.g., MAPK, Smad, Rho/Rho kinase) are included to gain a better understanding of overall effectors and effects of altered SMC signaling due to either genetic mutation or pharmacologic intervention. This rendering of the network was based on findings reported in over 100 archival papers covering diverse aspects of smooth muscle cell function (Table A in S1 Text) and visualized using Netflux (https://github.com/saucermanlab/Netflux) and the open-source software Cytoscape (v.3.8.2, cytoscape.org, [23]).

**Table 1:** Network model parameters.

Parameter	Value
Half-maximal activation	*EC*_50_ = 0.52
Hill exponential	*n* = 1.4
Intramural Stress, Shear Stress	*W* = 0.24054
Exogenous Angiotensin II, IFN-γ	*W* = 0.0
Oxygen, Cellular Energy	*W* = 0.5
Glucose, Leucine, Fibrillin	*W* = 0.25
Cell Receptor reactions	*W* = 0.85
Downstream reactions	*W* = 1.0

**Fig 2:**
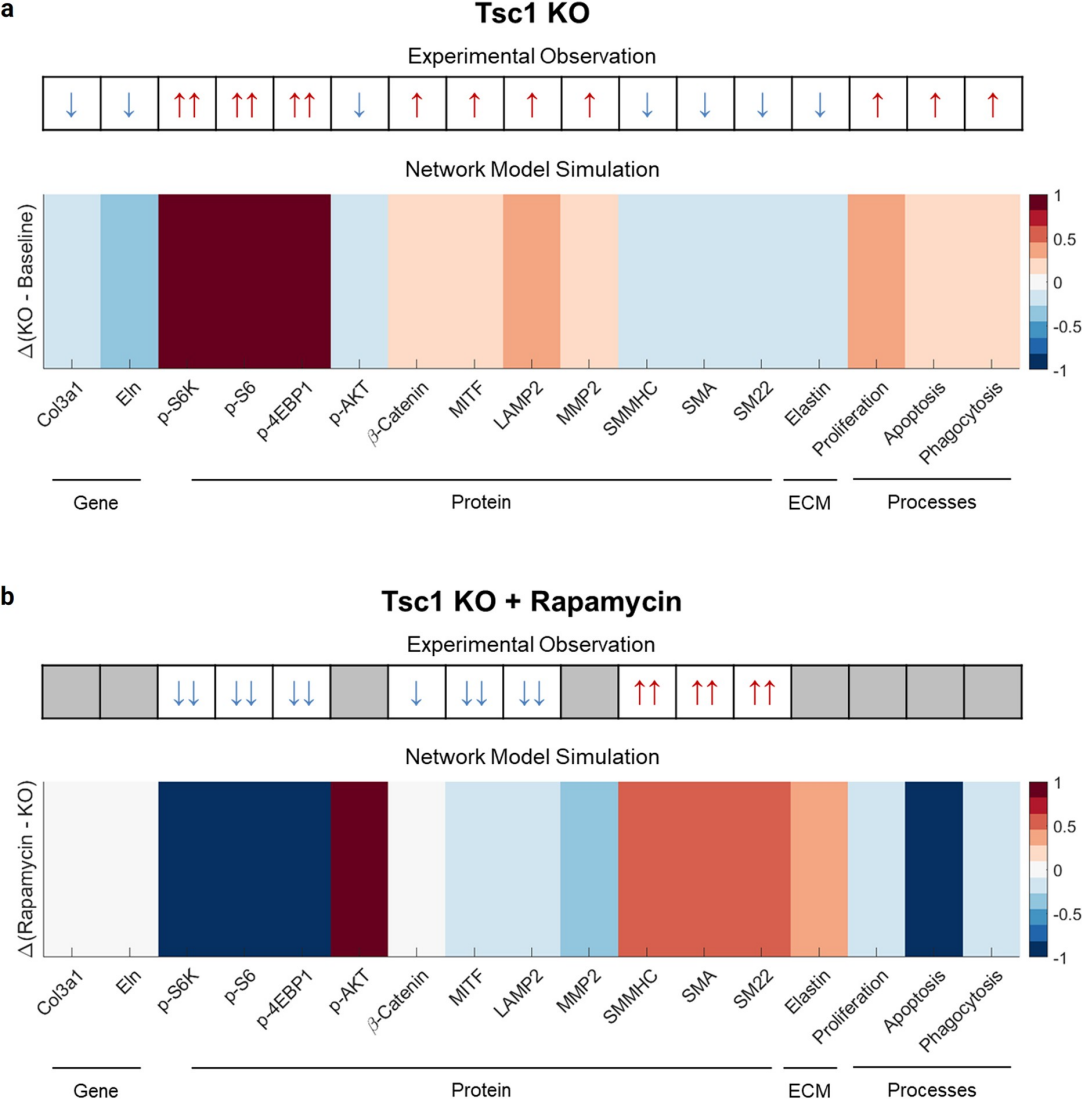
Qualitative comparison between network model simulations and experimental data reported by Li et al. [10]. a) Conditional *Tsc1* knock-out (KO) in aortic smooth muscle cells led to significant experimentally measured changes in various species at gene and protein (intracellular and extracellular matrix) levels, as well as increases in cell proliferation, apoptosis, and degradative activity. The signaling network model qualitatively captured all of these experimental changes between the KO and baseline cells. b) Addition of the mTORC1 inhibitor rapamycin to the KO cells led to decreases in mTORC1-associated species and lysosome-related species, as well as an increase in contractile protein expression. The signaling network model also qualitatively captured these experimental observations for rapamycin administration in the KO mice. See also S2 Fig.

As expected and desired, the model predicted that conditional SMC-specific KO of *Tsc1* (i.e., setting *y*_*max*_ = 0 for TSC1/2 in the network; Table 2) results in a hyperactivation of mTORC1 signaling, leading to upregulation and downregulation of multiple intracellular signaling molecules. Fig 2A qualitatively compares predicted results with those that were observed experimentally [10], focusing on increased or decreased activity of key species in the medial SMCs between Baseline and KO conditions. Species downstream of mTORC1, namely p-S6K, p-S6, and p-4EBP1, increased substantially in the KO, while upstream species such as p-AKT experienced only a moderate decrease. β-catenin and lysosome-related species, namely microphthalmia-associated transcription factor (MITF) and lysosome-associated membrane protein 2 (LAMP2), increased moderately due to the KO, as did the proteolytic matrix metalloproteinase-2 (MMP2) species. The contractile proteins smooth muscle myosin heavy chain (SMMHC), smooth muscle alpha actin (SMA), and smooth muscle SM22 decreased in the KO. Levels of ECM, here including the alpha 1 chain of type III collagen (*Col3a1*) and elastin (*Eln*) genes decreased moderately in expression in the KO and similarly the elastin protein decreased in the KO. The model thus matched qualitatively the changes in all 14 of the species reported experimentally.

**Table 2:** Summary of the three primary simulated conditions.

	Conditions		
	Baseline	KO	Rapa
TSC1/2 node *y*_*max*_	1.0	0.0	0.0
mTORC1 node *y*_*max*_	1.0	1.0	0.0

### Simulated rapamycin treatment qualitatively captures all observed changes in *Tsc1* KO aortas

Simulated treatment with the mTORC1 inhibitor rapamycin (Rapa) was modeled via a complete inhibition of the mTORC1 node (i.e., setting *y*_*max*_ = 0 for mTORC1 in the network; Table 2). Fig 2B shows changes in activation of key species between the KO and KO + Rapa with a qualitative comparison to experimental observations [10]. Again consistent with the experiments, the predicted activation of p-S6K, p-S6, and p-4EBP1 due to the KO decreased substantially with rapamycin treatment. The predicted activation of β-catenin decreased only slightly despite a significant decrease seen experimentally, while MITF and LAMP2 were predicted to decrease moderately, as seen experimentally. The contractile proteins SMMHC, SMA, and SM22 experienced substantial predicted increases in activation due to rapamycin, consistent with experimental findings. Overall, the model reproduced qualitatively the experimental changes in these 9 reported species. See, too, S2 Fig for a comparison of KO + Rapa to Baseline.

### Model of mTOR signaling qualitatively captures increased SMC proliferation, apoptosis, and degradative activity in *Tsc1* KO aortas

We used the activation levels of specific model species to estimate the level of proliferation, apoptosis, and degradative activity in *Tsc1* KO SMCs. Specifically, we let the mean of the activation levels of S6, β-catenin, and p38 reflect proliferation while the activation level of FOXO reflected apoptosis. Degradative activity was estimated based on the level of degraded elastin and activation of lysosomal LAMP1/2 proteins. Experimentally, *Tsc1* KO led to significantly increased proliferation but also apoptosis among SMCs in the aorta, as well as increased degradation of ECM components and degradative organelle activity in these KO cells. Our model qualitatively captured the increases seen in these cellular activities in the KO, as shown in Fig 2. Simulated rapamycin inhibition led to a subsequent decrease in these cellular activities.

### Network model predictions agree quantitatively with experimental findings

**Fig 3:**
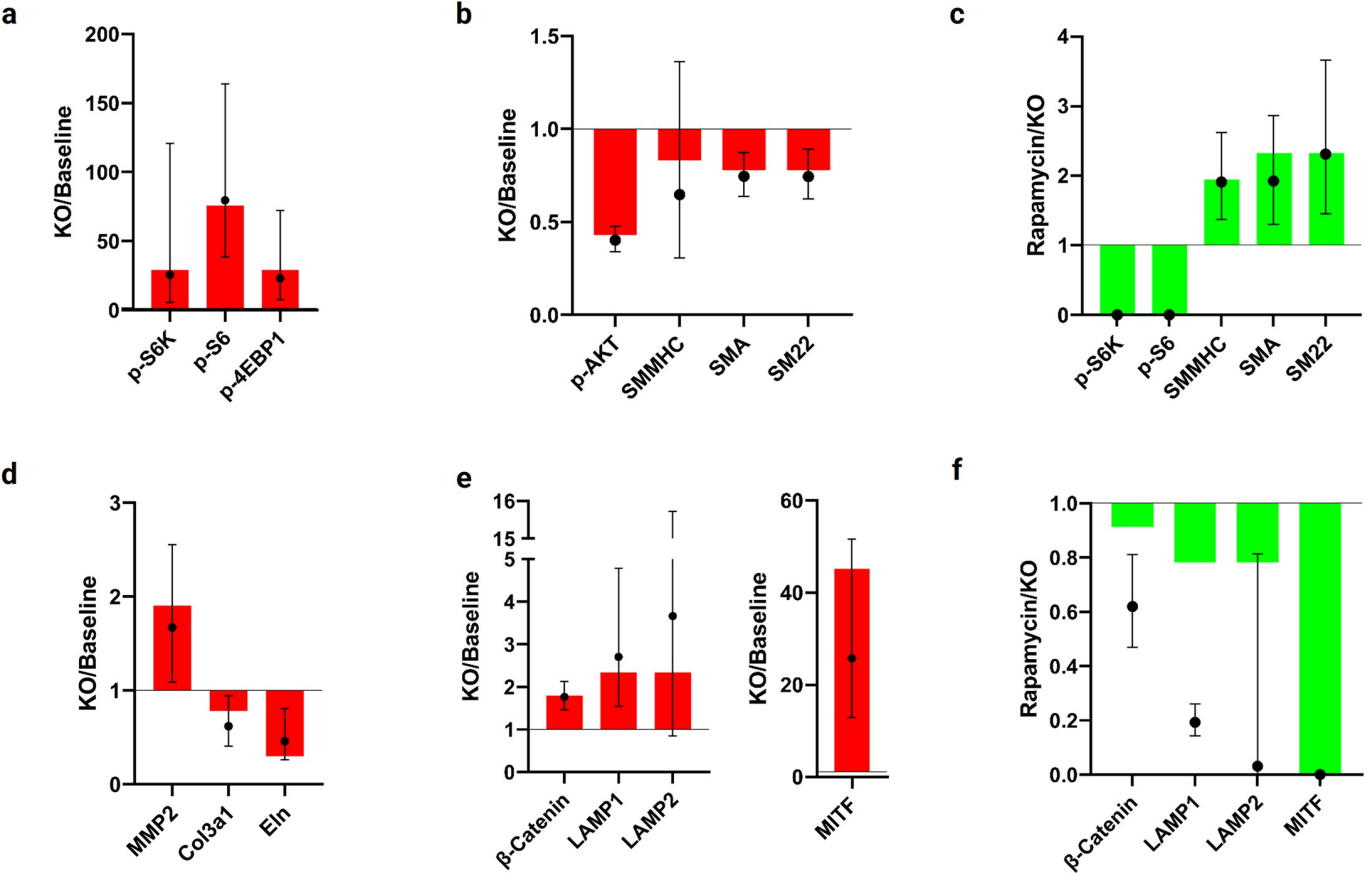
Quantitative comparison between network model results (bars) and experimental data (shown as point estimates (filled black circles) and 95% credible intervals (error bars) for the ratio of median expressions in KO versus baseline) reported by Li et al. [10]. a) The signaling network model quantitatively captured increases in expression of mTORC1-associated species (p-S6K, p-S6, and p-4EBP1) caused by *Tsc1* knock-out. b) The network model quantitatively captured the decrease in p-AKT and contractile proteins (SMMHC, SMA, and SM22) caused by *Tsc1* KO. c) Simulated inhibition of mTORC1 with rapamycin quantitatively captured the decrease in p-S6K and p-S6 activation and the decrease in contractile proteins observed experimentally. d) The network model quantitatively captured the increase in MMP2 expression and decrease in extracellular matrix transcripts *Col3a1* and *Eln* seen experimentally after *Tsc1* KO. e) The network model quantitatively captured the experimentally measured increases in lysosome-related proteins (β-catenin, LAMP1/2, and MITF) due to *Tsc1* KO. f) Inhibition of mTORC1 with rapamycin caused a decrease in lysosome-related species, which the network model captured qualitatively but not quantitatively. See Methods for details on the re-analysis of the experimental data from the literature.

Whereas logic-based models are well known to capture qualitative changes, we also sought to achieve quantitative agreements, where possible. Toward this end, we first reanalyzed the prior experimental findings [10], herein representing the data as ratios of median expressions with 95% credible intervals (see Methods and S3 Fig). Fig 3 compares results quantitatively both for the KO relative to baseline and for rapamycin treatment of the KO relative to untreated KO, with modeling predictions shown by the colored filled bars based on tuned parameters (S4 and S5 Figs) and the experimental data shown by the filled black circles and associated credible intervals. As it can be seen, the model predictions captured all quantitative changes well for the KO and many for rapamycin, though not well for β-catenin or LAMP1/2.

Specifically, note the hyperactivation of mTORC1 in the absence of TSC1/2 as evidenced by the activation of p-S6K, p-S6, and p-4EBP1 in both the KO model and the experiments relative to the baseline case. Our model predicted relative increases in activations of 29.31, 75.91, and 29.31 (Fig 3A), respectively, which fall within a 95% credible interval of the ratio of the medians in the experimental data (25.57 [5.44, 120.68], 79.57 [38.72, 163.80], and 23.06 [7.57, 72.04], respectively). These changes resulted directly from removal of the inhibition of mTORC1 by TSC1/2. The experimental data also showed a significant decrease in SMC contractile proteins in the KO mouse aortas relative to wild-type controls. We compared the relative activation of the key species SMMHC, SMA, and SM22 in the simulated KO model relative to the baseline model (Fig 3B), and found decreased activation ratios of 0.8313, 0.7780, 0.7780, respectively, all of which are within a 95% credible interval for the experimental data (0.6474 [0.3056, 1.3622], 0.7462 [0.6376, 0.8733], and 0.7440 [0.6242, 0.8918], respectively). The decrease in p-AKT, an upstream effector of mTOR signaling, was predicted to be 0.4300, which also compared well with experimental data (0.4022 [0.3395, 0.4770]). This change occurs because p-S6K inhibits activation of PI3K by IGFR and IRS-1 in a negative feedback loop (S1 Fig). Hyperactivation of p-S6K can thus lead to a substantial decrease in PI3K/AKT signaling and subsequent decreases in mTORC2 and RhoA. Both p-AKT and RhoA are involved in the activation of contractile proteins, thus accounting for their decreased levels. Simulating the early effects of rapamycin, in which we inhibited mTORC1, abolished the activation of p-S6K and p-S6, consistent with the experiments. The subsequent removal of the inhibitory effects of p-S6K (Fig 3C) led to increases in activation of the contractile proteins SMMHC, SMA, and SM22 (1.9391, 2.3204, and 2.3204, respectively). These increases fell within the 95% credible interval of the experimental data (1.9114 [1.3746, 2.6245], 1.9259 [1.3038, 2.8642], and 2.3107 [1.4538, 3.6662], respectively).

The SMCs in the *Tsc1* KO mice expressed decreased levels of synthetic markers, specifically transcripts associated with ECM production, relative to the control mice at 3 weeks following *Tsc1* KO. Our model predicted reduced activation levels of Col3a1 and Eln in the KO, with ratios of 0.7814 and 0.2988, respectively, relative to baseline (Fig 3D). The reduction in both species fell within a 95% credible interval of the ratio of experimental data medians (0.6195, [0.4065, 0.9443] and 0.4584 [0.2623, 0.8053], respectively). The predicted reduced expression in our model was due to disruption of TGFβR-TSC1-Smad2/3 signaling through TSC1/2 KO. Our model also captured changes in the proteolytic and lysosomal capabilities seen in degradative SMCs after *Tsc1* disruption (Fig 3D). The activation level of MMP2 increased in the KO relative to the baseline condition by a ratio of 1.9028, quantitatively similar to the behavior seen experimentally (1.6707, [1.0885, 2.5543]. Despite the decrease in activation of p-AKT, an effector of MMP2, we saw enhanced inhibition of GSK3 by p-S6K. GSK3 is a downstream protein in the Wnt-Frizzled signaling cascade and is responsible for inhibition of both β-catenin and MITF as well as activation of TSC1/2. Reduction in GSK3 activity was thus responsible for the predicted increases in the activation levels of β-catenin (1.7952) and the lysosomal-related proteins LAMP1, LAMP2, and MITF (2.3424, 2.3424, and 45.1490, respectively), as seen in Fig 3E, which all fell within a 95% credible interval of the experimental data (1.7620 [1.4623, 2.1244], 2.7029 [1.5385, 4.7815], 3.6597 [0.8488, 15.7193], and 25.7681 [12.8522, 51.6854], respectively). In the Rapa + KO simulation, we saw predicted decreases in activation levels of these lysosomal species (β-catenin: 0.9138, LAMP1: 0.7820, LAMP2: 0.7820, and MITF: 0). The model thus quantitatively captured the experimental behavior for LAMP2 and MITF (0.0312 [0.0012, 0.8134] and 0 [0, 0], respectively), but not for β-catenin or LAMP1 (0.6194 [0.4687, 0.8109] and 0.1931 [0.1432, 0.2613, respectively], as seen in Fig 3F.

### Classification of model species reveals distinct functional phenotypes in wild-type and *Tsc1* KO aortas

While SMCs have been traditionally described as having either a contractile or a synthetic phenotype, our prior study [10] revealed a third distinct (degradative) phenotype. We used our network model to understand better where within this expanded phenotypic space baseline and KO cells reside. For this purpose, each phenotype (contractile, synthetic, degradative) was described using a normalized activation, ranging from 0 to 1, which we calculated as the average activation of a set of key species (S6 Fig). The contractile phenotype was defined by SMMHC, SMA, and SM22; the synthetic phenotype was defined by Col3a1, Eln, and tissue inhibitors of metalloproteinases (TIMP); and the degradative phenotype was defined by LAMP2, MMP2, S6, MITF, and β-catenin. The baseline condition showed a balance between the contractile (0.5716) and synthetic (0.6848) phenotypes with little evidence of the degradative phenotype (0.1555), as seen in S6A Fig. The KO condition shifted this balance towards a degradative (0.5523) phenotype, with a decrease in the influence of both the contractile (0.4557) and synthetic (0.4620) phenotypes (S6B Fig).

### Distinct proteomic clusters arise in simulated wild-type and *Tsc1* KO SMC populations

**Fig 4:**
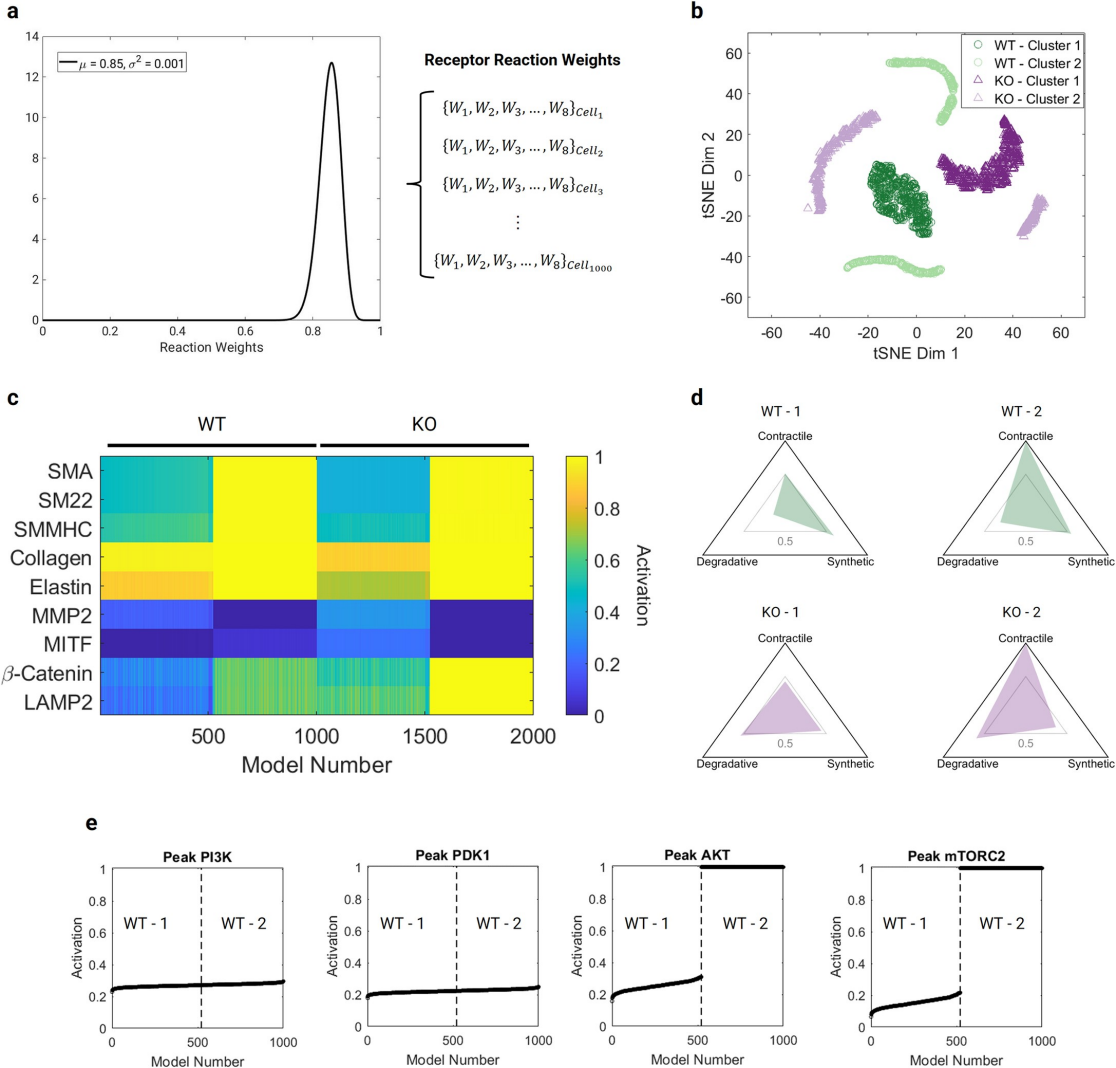
Cell population simulations. a) A beta distribution, with μ = 0.85 and σ^2^ = 0.001, was used to randomly sample receptor reaction weights for 1000 different signaling network models for wild-type and KO cells. Each of the 7 receptor reactions in a distinct cell was assigned a different weight. b) Visualization of wild-type (circles) and KO (triangles) clusters using a t-distributed stochastic neighbor embedding (tSNE) algorithm. Two primary clusters for each condition were found using a density-based spatial clustering of applications with noise (DBSCAN) algorithm for the steady-state activation levels of network species. c) Heatmap showing the activation level of species relevant to contractile, synthetic, and degradative phenotypes for each of the 1000 models in each population (WT and KO). Clusters WT– 1 and KO– 1 are characterized by moderate-to-low activation of contractile proteins (SMA, SM22, SMMHC), while clusters WT– 2 and KO– 2 both have saturated levels of these species. d) The phenotypic characteristics of the average cell in each cluster was visualized in the contractile-synthetic-degradative phenotypic space. Both KO clusters are more degradative than the WT clusters, but clusters WT– 2 and KO– 2 retain their contractile capacity. e) The peak activation of species in the PI3K/AKT/mTORC2 positive feedback loop of WT models show that beyond a threshold (0.2716) of PI3K activation, the level of AKT and mTORC2 saturates, causing a bistable division into distinct clusters.

We created a heterogenous population of aortic SMC network models by randomly specifying individual receptor reaction weights for each cell using a beta distribution of mean = 0.85 and variance = 0.001; this distribution was characterized by parameters α = 107.525 and β = 18.975 (Fig 4A). We again defined a subset of species relevant to the contractile, synthetic, and degradative SMC phenotypes, namely {SMA, SM22, SMMHC}, {collagen, elastin, MMP2}, and {MITF, β-catenin, and LAMP2}, and used the activation level of these species to identify distinct proteomic clusters within the simulated cell populations. Running a DBSCAN algorithm on the combined baseline and KO cells revealed 4 clusters, two corresponding to the wild-type cells (WT– 1, WT– 2) and two to the KO cells (KO– 1, KO– 2). Fig 4B visualizes the clusters using a t-distributed stochastic neighbor embedding (tSNE) algorithm, including the DBSCAN classification. In Fig 4C, we show a heatmap of the activation level of each relevant species for every model, divided into their corresponding clusters. The heatmap shows that clusters WT– 2 and KO– 2 are characterized by saturated levels of the contractile proteins SMA, SM22, and SMMHC. Cluster WT– 1 has moderate levels of the contractile proteins, while cluster KO– 1 shows a decrease in these species compared to WT– 1. When we consider the average phenotype for cells within each cluster, shown in Fig 4D, we see that clusters WT– 2 and KO– 2 are both highly contractile, regardless of the effect of TSC1/2 and mTORC1 signaling. Both clusters KO– 1 and KO– 2 show a higher degradative phenotype than either of the wild-type clusters. Because the PI3K/AKT/mTOR signaling pathway influences contractile protein expression, we looked further into the activation level of key species within this pathway for the cell population. Fig 4E shows peak activation levels of PI3K, PDK1, AKT, and mTORC2 for each cell in the baseline condition. Both PI3K and PDK1 increase smoothly, while AKT and mTORC2 saturate for all models in cluster WT– 2, revealing a threshold for PI3K activation (0.2716) that triggers AKT saturation in these cells.

### Sub-network analysis reveals bistable behavior in the PI3K/AKT/mTOR signaling pathway

**Fig 5:**
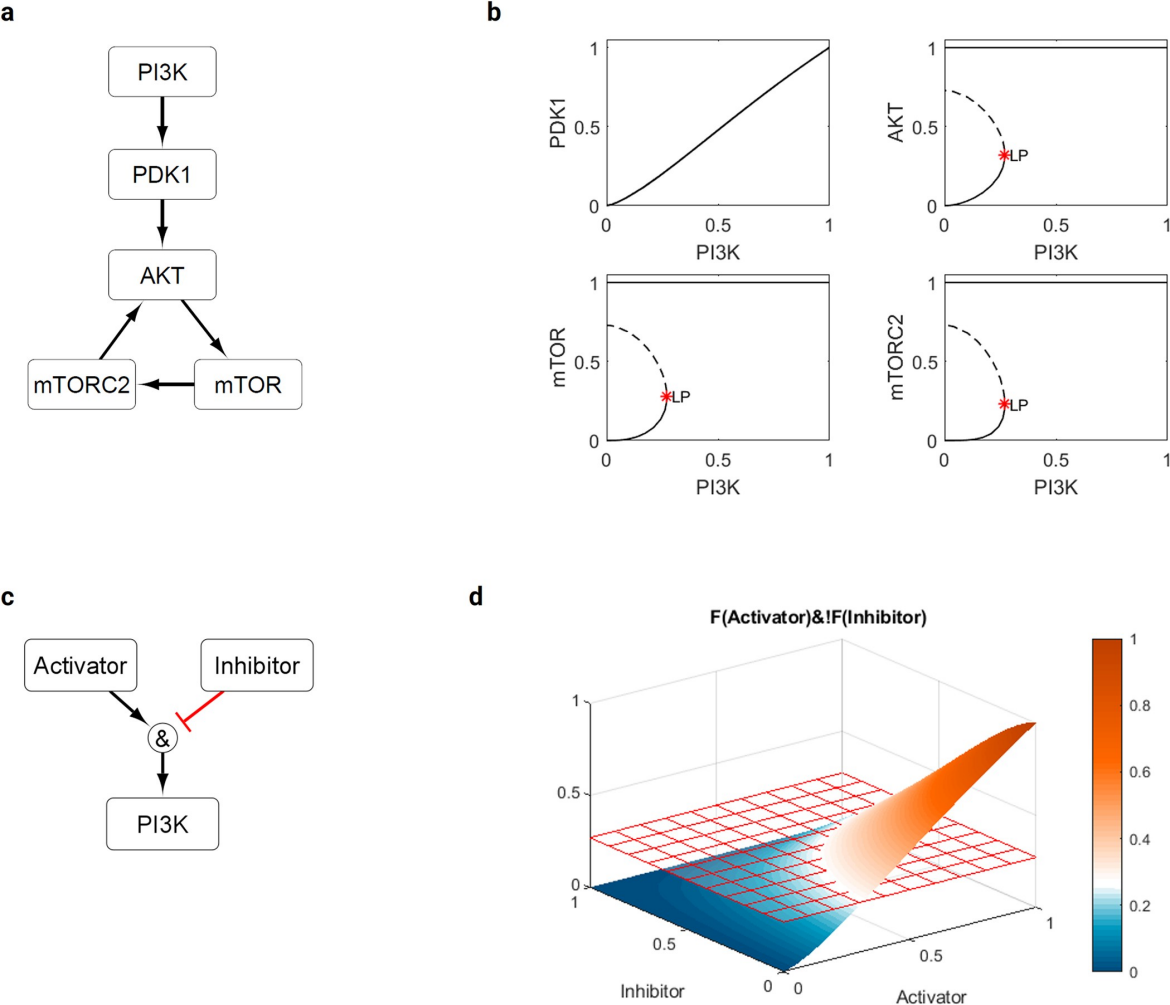
Focus on the PI3K/AKT/mTOR signaling sub-network structure. a) Simplified network diagram showing the positive feedback loop for AKT activation with PI3K serving as an input. b) MatCont bifurcation analysis showing the stable (solid lines) and unstable (dashed lines) steady-state solutions for each downstream species in the model shown in (a) for different levels of PI3K input. While there is no bifurcation for PDK1, the other three species in the network (AKT, mTOR, and mTORC2) exhibit a limit point bifurcation (LP), indicated by a star, detected at a level of PI3K = 0.27083. c) Simplified network diagram showing an activator and inhibitor pair interacting via an AND gate to activate PI3K. d) Surface plot showing the steady-state activation level of PI3K for different levels of an upstream activator and inhibitor pair. The threshold for saturation of the downstream species in the positive feedback loop is shown as a grid. Increasing the level of inhibitor prevents PI3K from crossing this threshold.

Finding two distinct clusters in each condition in the population studies, even though receptor reaction weights varied smoothly within a unimodal distribution, led us to investigate further the role of feedback loops in PI3K/AKT/mTOR signaling. A known possible consequence of positive feedback loops is bistability (the co-existence of two stable states) and the presence of so-called bistable switches, which describe sudden transitions between these states at threshold values [24,25]. Bistability thus became a candidate mechanism for the behavior we observed. We isolated a simplified sub-network with five species (PI3K, PDK1, AKT, mTOR, mTORC2), including the positive feedback loop (Fig 5A), using PI3K as the input. To understand the dynamics of this sub-network, we ran a MatCont bifurcation analysis to generate one-parameter bifurcation diagrams for the four downstream species (Fig 5B), where stable steady states are shown by solid lines as a function of PI3K value. While PDK1 did not exhibit any bifurcations, the species in the positive feedback loop exhibited limit point bifurcations (also known as saddle-node or fold bifurcations) at a PI3K level of 0.27083 and featured bistable behavior below this threshold. In the bistable region, the stable steady-state solution will settle at either a low or a saturated level even at low PI3K steady-state values, depending on the initial level of each species relative to the dashed unstable branch. If beginning on the lower stable branch, an increase in PI3K activity through the limit point bifurcation will lead to a switch to the saturated state, which is self-sustaining and irreversible assuming there are no changes to the network structure or Hill parameters. This threshold of ~0.27 for the sub-network is consistent with that detected in the aforementioned analysis of the full network (Fig 4E), suggesting that sub-networks can be used to study underlying dynamics. Moreover, the isolated positive feedback loop is sufficient to create the observed behavior, suggesting that it is the key mechanism. Because PI3K is attenuated by S6K signaling in the full network (S1 Fig), we also sought to understand the influence of both activation and inhibition on the bistable system (Fig 5C). The steady-state activation of PI3K and its ability to cross the threshold depends on a balance of activation and inhibition, as shown in Fig 5D. Increasing the level of inhibition prevents PI3K from crossing the threshold and triggering saturation of AKT signaling.

## Discussion

Whereas we built an aortic SMC signaling model that incorporates many of the key signaling pathways, we necessarily focused on the mTOR pathway given the availability of detailed data [10] and the continuing demonstration that rapamycin has proven remarkably effective in rescuing the in vivo aortic phenotype in many murine models of aortopathy. In particular, rapamycin has proven effective in attenuating medial degeneration and associated aneurysmal enlargement or dissection in elastase models [26–28], angiotensin II infusion models [29,30], β-aminopropionitrile (BAPN) induced models [31,32], and genetically triggered models, including those affecting transforming growth factor-β signaling [8], fibrillin-1 [33], and mTOR hyperactivation [10], among others. Indeed, a comprehensive proteomics study of the *Fbn1*^*C1039G/+*^ mouse model of Marfan syndrome revealed mTORC2 associated rictor as a key signaling target [34]. Reported mechanisms by which rapamycin is protective are many, including reduced inflammatory cell infiltration (neutrophils and macrophages), reduced cytokine activity (interleukin-1β and interferon-γ), reduced matrix metalloproteinase activity (MMP2, 9), and increased SMC contractile proteins (SMA and SMMHC). Maintenance of actomyosin activity is critical both for facilitating appropriate SMC mechanosensing [6] and reducing wall stress on a structurally vulnerable aortic wall [35].

Whereas it has long been known that rapamycin inhibits SMC proliferation/migration [36,37] while promoting a contractile SMC phenotype [38,39], less is known about its direct effects on ECM turnover. Nevertheless, data suggest that rapamycin can reduce accumulation of hyaluronan [20] and collagen [21,40], both key constituents of aortic remodeling, particularly in cases of compromised elastic fiber integrity, a common characteristic of thoracic aortopathy. Indeed, studies in tendons suggest that signaling via *β*_1_ integrin subunits through integrin linked kinase (ILK) drives collagen synthesis via AKT/mTOR signaling [41]. Mouse models of *Ilk* deletion present with thoracic aneurysms [42,43], perhaps suggesting yet another role of compromised mechanosensing and inappropriate maintenance or remodeling of ECM. Such changes in matrix turnover must be considered carefully, however, for structural functionality results not just from secretion, but also post-translational modifications and organization of matrix that affect fiber size, undulation, orientation, and cross-linking [44,45]. The present model cannot predict post-translational changes that include fibrillogenesis or cross-linking, hence highlighting one area of future need.

Notwithstanding the complexity of intracellular signaling networks (Fig 1), it is remarkable that logic-based models can often capture qualitative findings well with uniform values of the key parameters (*n*, *EC*_50_, and multiple weights *W* –see Methods; Table 1), as noted previously by others [46]. We found that uniform parameter values not only provided good qualitative agreements with data (Fig 2), they also yielded surprisingly good quantitative agreement in many cases (Fig 3). This is remarkable but engenders confidence in exploring predictions associated with different values of parameters. Such simulations appear to be particularly useful given that single-cell RNA sequencing reveals considerable variability across otherwise similar SMCs in both health and disease, including Marfan syndrome [47] and mTOR hyperactivation [10].

We found that simple perturbations in specific parameters, such as the weight of receptor reactions, can cause distinct cell proteomic clusters to emerge. Our findings suggested further that the balance of positive and negative feedback loops in the PI3K/AKT/mTOR pathway was particularly critical to the emergence of these simulated clusters. Unexpectedly, we found that the positive feedback loop between AKT and mTORC2 led to a bistable switch in the network, with either high or low levels of AKT and contractility dependent on the peak activation level of PI3K. The negative feedback loop between mTORC1-S6K and PI3K reduced the peak PI3K activation level, providing some self-regulation against the switch to a highly contractile state. Of note, the balance between positive and negative feedback can be controlled pharmacologically; for example, mTORC1 inhibition by rapamycin disrupts the negative feedback. Other computational studies have discussed the possibility and importance of bistability in mTOR signaling [48–50]. In the setting of aortic mechanobiology, the presence of a bistable switch in PI3K/AKT/mTOR signaling could be particularly important given the downstream impact on contractile protein expression, and further experimental studies investigating potential bistability could be important to better understand and treat thoracic aortopathies. In their 2020 study, Li et al. [10] found many distinct transcriptomic clusters, including some with upregulated or downregulated contractile protein transcripts, using single-cell RNA sequencing. It was beyond the present scope, however, to clarify the differences between clusters using our model, as we instead focused on the in vivo phenotypes.

Future modeling must address current limitations, including the lack of cell-cell interactions, ECM ligand-dependent signaling, and coupling across scales from cell-to-tissue. That is, there is a need to couple the present cell signaling model with a tissue level model of the evolving aortic geometry, composition, properties, and function, as achieved previously for angiotensin II induced hypertension [16]. Such cell-tissue level coupling provides another important level of feedback. Additionally, future studies can include uncertainty quantification to understand better the impact of parameter variability within the network. Novel methods to account for both data and parameter uncertainty in network models are continuing to emerge [51], as are computational tools to facilitate their practical application [52]. Moreover, we have recently developed an uncertainty quantification pipeline for estimating local mechanical properties of the vessel wall [53], thus paving the way to addressing both tissue-level and (sub)cellular uncertainties in mechanobiological metrics within a future multiscale modeling framework. Nevertheless, the present cell signaling network represents the first SMC-specific model having both qualitative and quantitative utility in describing and predicting emergent characteristics seen in the thoracic aorta, here for the *Tsc1* KO mouse. We found that, even when using uniform values for the logic-based model parameters (*EC*50, *n*, *W*), a single network captured salient aspects of the effect of both mTOR hyperactivation and pharmacologic rescue with rapamycin, with perturbations that impact PI3K/AKT/mTOR signaling leading to different cell phenotypes due to a bistable switch caused by positive feedback within this signaling pathway.

## Methods

### Logic-based modeling

Cell signaling networks can be modeled using either reaction kinetics or logic-based approaches. We employed the latter, motivated in large part by demonstrated successes in modeling cardiac cell signaling [46]. Briefly, the continuous logic-based approach that we employ [54] results in a system of ordinary differential equations (ODEs) in time that describe interactions (edges) amongst the different molecular species (nodes). The ODEs are based on normalized Hill-type functions [55] that represent activation (*act*) or inhibition (*inhib*) of each species in the network [46,54,56,57], illustrated in the equations below:

$$f_{act}(X)=\frac{\beta X^{n}}{K^{n}+X^{n}}$$

$$f_{inhib}(X)=1-\frac{\beta X^{n}}{K^{n}+X^{n}}$$

with,

$$\beta =\frac{{EC}_{50}^{n}-1}{2{EC}_{50}^{n}-1}\;\mathrm{and}\;K^{n}={\left(\beta -1\right)}^{\frac{1}{n}}.$$

These functions depend on the half-maximal activation (*EC*_50_) and Hill exponent (*n*), which can be tuned to obtain proper model behavior. In particular, each species in the cell signaling network is represented by a node with a normalized activation level ranging from 0 to 1, with reactions between species defined using AND and OR logic-gates. Activation using an AND gate requires the interaction of two or more upstream species. For example, let the activation of species *C* (*y*_*C*_) depend on the combined activation of species *A* (*y*_*A*_) and *E* (*y*_*E*_), namely

$$\frac{dy_{C}}{dt}=\frac{1}{\tau_{C}}(AND(y_{A},y_{E})y_{C,max}-y_{C})$$

with,

$$AND=W_{AEC}f_{act}(y_{A})f_{act}(y_{E}).$$

Meanwhile, OR gates allow activation by multiple upstream species independently, as shown in below for the activation of species *D* (*y*_*D*_) by either species *B* (*y*_*B*_) or *E* (*y*_*E*_).

$$\frac{dy_{D}}{dt}=\frac{1}{\tau_{D}}(OR(y_{B},y_{E})y_{D,max}-y_{D})$$

with,

$$OR=W_{BD}f_{act}(y_{B})+W_{ED}f_{act}(y_{E})-W_{BD}f_{act}(y_{B})W_{ED}f_{act}(y_{E}).$$

The degree to which a reaction can activate or inhibit a species depends on its specified weight (*W*), which also can range from 0 to 1. The maximum activation (*y*_*max*_) of and time constant (*τ*) for each species are set to a default value of 1 for all species in this model, although the former can be altered to simulate a knock-down (value less than 1) or knock-out (value 0).

### Model parameterization and *Tsc1* KO simulations

The primary parameters within the model are thus the two Hill parameters (*n* and *EC*_50_) and individual reaction weights (*W*). External inputs also have weight-like parameters that represent their normalized magnitude. Given the exquisite mechano-sensitivity of vascular cells, we allow pressure-induced intramural stress (*W* = 0.24054), flow-induced wall shear stress (*W* = 0.24054), exogenous angiotensin II and IFN-γ (though absent here, thus *W* = 0), oxygen/cellular energy (*W* = 0.5), and other inputs (*W* = 0.25) as external stimuli (see Fig 1). Extensive initialization studies revealed the following preferred values: *n* = 1.4, *EC*_50_ = 0.52, *W* = 0.85 for receptor reactions, and *W* = 1 for “downstream” reactions. That is, we tuned the Hill parameters, input weights, and receptor reaction weights such that model-predicted ratios for the mTORC1-associated species and contractile proteins between conditions, as described below, would fall within a 95% credible interval of the experimental data here taken from [10]. One advantage of logic-based models is that uniform parameter values often yield good predictions, hence for simplicity and without a loss of generality, these parameters were applied uniformly for the 81 overall species and 138 overall reactions that are seen in Fig 1. Table 1 summarizes the model parameters used. We ran all simulations using MATLAB (R2020a, Mathworks).

Once parameterized, the model was used to simulate three conditions (Table 2): normal signaling in wild-type control C57BL/6 SMCs (Baseline), postnatal SMC-specific knock-out of *Tsc1* (KO), and postnatal SMC-specific knock-out of *Tsc1* with rapamycin treatment (Rapa). We simulated the *Tsc1* KO by setting the *y*_*max*_ value of the TSC1/2 node to 0 while keeping all other parameters constant. For the Rapa simulation, we additionally set the *y*_*max*_ value of mTORC1 to 0, corresponding to full inhibition by rapamycin within the early period following the initiation of treatment. We then used the ratios of steady-state activation levels of relevant model species to compare our model behavior with the experimental data [10].

### Reanalysis of experimental data

Central to assessing our signaling network model was a meaningful comparison of predictions with experimental data. To this end, our objective was to evaluate the extent to which the tuned model was able to quantitatively capture altered expressions of species of interest following both *Tsc1* KO and mTORC1 inhibition by rapamycin (Fig 3). Although the relevant data had been analyzed previously to identify significant differences in relative expression between experimental groups [10], we reanalyzed the experimental data to quantify the ratio of KO and baseline as well as treated (rapamycin) and untreated KO group expression levels for each species of interest. To be most representative of the observed data, the tuned model should predict values close to the central tendency of this ratio of group-wise expressions. We therefore chose to compare the model predictions to the *ratio of median expression levels* between groups—for example, the ratio of a species’ median expression in the KO condition and its median expression in the baseline condition. Because the true (i.e., population) distributions of expression levels are unknown, the median expression levels must be estimated for each group using the experimental data; thus, the ratio of these median values is itself an estimated quantity with an associated uncertainty. In Fig 3, for each ratio shown, we report a point estimate (filled black circle) as well as a 95% credible interval (bars) for this quantity, computed using the following methodology.

For example, let *Y*_Base_ and *Y*_KO_ be the relative expressions of a hypothetical species of interest in the baseline and KO groups, respectively, measured via western blot densitometry and normalized with respect to a loading control (S3A Fig). Under the assumption that the strictly positive expression levels within each group are lognormally distributed, the log-transformed expression levels ln(*Y*_*i*_), which are unbounded (S3B Fig), can be modeled using a normal distribution. Adopting a Bayesian approach as we have done previously [53], for an uninformative prior proportional to the reciprocal of the variance, the posterior marginal distributions of the group-specific median log-expressions *μ*_Base_ and *μ*_KO_ are non-standardized Student’s *t* distributions (S3C Fig). The difference between these, *δ* = *μ*_KO_−*μ*_Base_, is distributed according to the appropriate convolution of their individual distributions (S3D Fig). For probability density functions *f*_Base_(*μ*_Base_) and *f*_KO_(*μ*_KO_),

$$f_{\delta }(\delta )=\int_{-\infty }^{\infty }f_{KO}(\mu_{KO})f_{Base}(\mu_{KO}-\delta )d\mu_{KO}.$$

Inverting the earlier log-transformation yields

$$exp(\delta )=exp(\mu_{KO}-\mu_{Base})=\frac{exp(\mu_{KO})}{exp(\mu_{Base})},$$

which is the desired ratio of group-wise median expression levels. Noting that quantile order is preserved under monotonically increasing transformations, the point estimate and equi-tailed credible interval bounds for exp(*δ*) are computed directly by exponentiating the 2.5^th^, 50^th^, and 97.5^th^ percentiles of *δ* (S3E and S3F Fig).

### Network parameter sensitivity

Another critical step in building our network model was tuning the parameters such that we could obtain a quantitative match to the experimental data [10], specifically the increase in species downstream of mTORC1 (p-S6K, p-S6, and p-4EBP1) and the decrease in p-AKT and contractile proteins (SMMHC, SMA, SM22). To achieve this, we tuned the model using a parameter sweep [57] of: the Hill parameters (1) *EC*_50_ and (2) *n*, (3) the input weight of pressure-induced intramural stress and wall shear stress, (4) the input weight of oxygen and cellular energy, (5) the weight of other inputs (Glucose, Leucine, Fibrillin), (6) the weights of the receptor reactions, and (7) the downstream reaction weights. The exogenous angiotensin II input weight was kept at 0 because we did not simulate any experiments where angiotensin II was administered exogenously. While the parameters were tuned in parallel, we found the sensitivity of representative species of interest to different parameter ranges. S4 Fig shows the solutions that fell within a 95% credible interval, as described above, for each of our species of interest within a two-dimensional solution space for the following combinations of parameters: *EC*_50_ and *n* (S4A Fig), receptor reaction weights and downstream reaction weights (S4B Fig), and the weight of input oxygen and cellular energy and weight of the remaining model inputs (Glucose, Leucine, Fibrillin, S4C Fig). We see in S4 Fig that p-AKT and p-S6 are the most sensitive species, while SMMHC was not sensitive to these parameters over the ranges explored. Finally, note the effect of the mechanics-based inputs, intramural stress and wall shear stress, on the KO/baseline ratios of our relevant species, shown in S5 Fig. As with the other parameters, there is a narrow range of input weights for which p-AKT and p-S6 fell within their respective 95% credible intervals.

### Population simulations

While the tuned network model represents an average aortic SMC, in an actual vessel we would expect to find a heterogeneous population of SMCs that need not respond collectively to particular perturbations. Thus, we used our network model to simulate distinct SMCs within a population, not unlike that revealed by single cell RNA sequencing. One advantage of these fast network models is the ability to run large numbers of models (i.e., cells) efficiently. Thus, using the same network architecture and Hill parameters (*EC*_50_, *n*), we created a heterogeneous cell population by varying only the receptor activation reaction weights based on random sampling of a beta distribution with a mean of 0.85 and a variance of 0.001, with corresponding values α = 107.525 and β = 18.975 characterizing the distribution. Each receptor reaction in the cell was assigned a distinct weight, and a total of 1000 cells were simulated. For each cell, we ran the baseline and KO conditions, as described above. We then used a subset of network species relevant to the three primary phenotypes (contractile, synthetic, degradative) to run a density-based spatial clustering of applications with noise (DBSCAN) algorithm on the population to obtain distinct proteomic clusters. For the DBSCAN algorithm, we set the maximum radius (epsilon) = 0.25 and the minimum points threshold (MinPts) = 25, based on preliminary sensitivity analyses of the parameters and comparison against principal component analysis with k-means clustering of additional test cases, with a silhouette analysis to obtain the optimal cluster number.

### Simplified PI3K/AKT/mTOR signaling network

Motivated by results from the full model, we also studied a simplified sub-network focused only on the PI3K/AKT/mTOR signaling pathway to understand better how possible positive and negative feedback interactions could lead to bistable behavior. We included 5 species (PI3K, PDK1, AKT, mTOR, and mTORC2) and 5 reactions. This simplified network included the positive feedback loop between AKT and mTORC2, and PI3K served as the input. We performed numerical continuation and bifurcation analyses using the open-source software MatCont (https://sourceforge.net/projects/matcont/, [58]) through MATLAB. This analysis tracks equilibrium points of a system of ODEs given changes in a particular bifurcation parameter, which in our case was the input activation level of PI3K. MatCont solves both stable and unstable equilibria, allowing us to identify and classify any bifurcations that might occur for each species in the network.

Bifurcation analysis is used to determine qualitative changes in model behavior as a function of model parameters. Most commonly, changes in the existence and stability of steady states (equilibria) are of interest. For continuous systems of ODEs defined by $\dot{x}=f(x)$, the steady states are solutions of *f*(***x***) = **0** and the stability of these steady states are determined by the eigenvalues of the Jacobian matrix evaluated at this point. Briefly, this is motivated by considering a small perturbation to a given equilibrium solution and linearizing the system via a first-order Taylor series approximation. The eigenvalues show whether the perturbation would shrink (negative real parts) or grow (positive real parts), and can thus be used to classify the point as stable or unstable, respectively.

To track equilibria as a function of a given model parameter *α* (known as the bifurcation parameter), numerical continuation is used to follow solutions of *f*(***x***, *α*) = **0**. We used the numerical continuation software Matcont for this analysis, which is freely available at https://sourceforge.net/projects/matcont [58] and runs through MATLAB. Branches of the stable and unstable equilibrium solutions are followed using a prediction-correction continuation algorithm, and eigenvalues are evaluated along solution branches to find and classify bifurcation points. For example, limit point bifurcations (as observed in Fig 5) occur when one eigenvalue has a zero real part. To generate bifurcation diagrams, the user inputs the model equations as well as initial equilibrium points, from which one specifies a forward or backward evolution of the bifurcation parameter.

The governing equations for our simplified 5-species sub-network (Fig 5A) are

$$\frac{dPDK1}{dt}=\frac{\beta {PI3K}^{n}}{K^{n}+{PI3K}^{n}}-PDK1,$$

$$\frac{dAKT}{dt}=\frac{\beta {PDK1}^{n}}{K^{n}+{PDK1}^{n}}+\frac{\beta {mTORC2}^{n}}{K^{n}+{mTORC2}^{n}}-\frac{\beta {PDK1}^{n}}{K^{n}+{PDK1}^{n}}\frac{\beta {mTORC2}^{n}}{K^{n}+{mTORC2}^{n}}-AKT,$$

$$\frac{dmTOR}{dt}=\frac{\beta {AKT}^{n}}{K^{n}+{AKT}^{n}}-mTOR,$$

$$\frac{dmTORC2}{dt}=\frac{\beta {mTOR}^{n}}{K^{n}+{mTOR}^{n}}-mTORC2,$$

where *PI*3*K* is the bifurcation parameter and

$$\beta =\frac{{EC}_{50}^{n}-1}{2{EC}_{50}^{n}-1},$$

$$K^{n}={\left(\beta -1\right)}^{\frac{1}{n}},$$

as described above. As in the full network, we use *w* = *y*_*max*_ = *τ* = 1 (which have thus been omitted in the governing equations) and *n* = 1.4 and *EC*_50_ = 0.52.

Steady states, where *f*(***x***, *PI*3*K*) = **0**, are given by

$$PDK1=\frac{\beta {PI3K}^{n}}{K^{n}+{PI3K}^{n}},$$

$$AKT=\frac{\beta {PDK1}^{n}}{K^{n}+{PDK1}^{n}}+\frac{\beta {mTORC2}^{n}}{K^{n}+{mTORC2}^{n}}-\frac{\beta {PDK1}^{n}}{K^{n}+{PDK1}^{n}}\frac{\beta {mTORC2}^{n}}{K^{n}+{mTORC2}^{n}},$$

$$mTOR=\frac{\beta {AKT}^{n}}{K^{n}+{AKT}^{n}},$$

$$mTORC2=\frac{\beta {mTOR}^{n}}{K^{n}+{mTOR}^{n}},$$

from which two steady state solutions are the zero state

[PI3K,PDK1,AKT,mTOR,mTORC2]=[0,0,0,0,0]

and the saturated state

[PI3K,PDK1,AKT,mTOR,mTORC2]=[1,1,1,1,1].

These solutions were used to begin the continuation, with equilibria followed forwards from the zero state and backwards from the saturated state. The range under investigation was restricted to [0,1] for all species.

The *PDK*1 stable branch had no limit point bifurcation (Fig 5B), and the same solution was obtained from both the forward and backward evolutions. The remaining species had distinct upper and lower branches, with the lower stable and unstable branches obtained from the forward evolution, and the upper branch from the backward evolution. The coexistence of two stable equilibria for some values of PI3K demonstrates bistability in the system, with the final solution depending on the initial condition.

Note finally that we held other model parameters (*n* and *EC*_50_) constant to enable a one-parameter analysis, but the position of the limit point bifurcation on the lower branch depends also on these values (S7 Fig). This dependency occurs since the Hill parameters regulate signal transmission and the strength of the positive feedback.

## Supporting information

S1 Fig Schematic rendering of the smooth muscle cell network model (cf. Fig 1, main text), but with an emphasis on the embedded PI3K/AKT/mTOR signaling pathway. The solid ellipse highlights the TSC1/2 node, which we used to simulate Tsc1 knock-out by setting its *y*_*max*_ = 0. The dashed ellipse highlights the mTORC1 node, which is the primary target of the inhibitor rapamycin. We simulated full inhibition by rapamycin by setting *y*_*max*_ = 0 for the mTORC1 node. See Table A in S1 Text and the associated 105 references that were used to build the overall network. A solid black line indicates activation; a red dashed line indicates inhibition.
(TIF)

S2 Fig Heatmap showing the change in activation between the simulated inhibition of mTORC1 signaling with rapamycin and the baseline model. While the experimental data from Li et al. (2020) did not include this comparison, we have added it for completeness. Rapamycin leads to greater activation of p-AKT and contractile signaling, as well as reductions in apoptosis, matrix transcripts, and MMP2 compared to the baseline model.
(TIF)

S3 Fig Experimental data reanalysis pipeline. a) Relative expression data for a hypothetical species derived from western blot densitometry normalized to a loading control, with baseline (Base) and *Tsc1* null (KO) groups each having a sample size of 4. b) Log-transformation of the relative expression data in (a). c) Posterior distributions for the group-specific median log-expressions *μ*_Base_ and *μ*_KO_, assuming the log-expression data within each group are normally distributed (i.e., assuming the untransformed data are lognormally distributed). d) Posterior distribution for the difference in median log-expressions, *μ*_KO_−*μ*_Base_. e) Point estimate and 95% equi-tailed credible interval for the difference in median log-expressions. f) Point estimate and 95% equi-tailed credible interval for the KO/Baseline ratio of median expressions. The point estimate and interval results shown for all species in Fig 3 correspond to those shown in (f), computed via the methodology presented in Methods.
(TIF)

S4 Fig Two-dimensional solution space masks for combinations of two parameters. Blue regions indicate combinations where the solution falls within a 95% credible interval of data for the species of interest. The star corresponds to the parameters used in the network model. Combinations of parameters: a) *EC*_50_ and *n*; b) receptor reaction weights and downstream reaction weights; and c) the weight of input oxygen and cellular energy and weight of the remaining model inputs (Glucose, Leucine, Fibrillin).
(TIF)

S5 Fig Effect of mechanical inputs on the *Tsc1* KO/baseline ratio. The pressure-induced intramural stress and wall shear stress are kept equal in our simulations. In each panel, the blue line corresponds to the *Tsc1* KO/baseline ratio for each species, with the solid and dashed black lines showing the point estimate and 95% credible interval for the KO/baseline ratio of median expressions, based on the experimental data. The star indicates the stress parameters used in the network model.
(TIF)

S6 Fig Visualization of the baseline (WT) and *Tsc1* null (KO) results within a smooth muscle cell phenotypic space bounded by vertices defined as purely contractile (1.0), purely synthetic (1.0), or purely degradative (1.0). The degree of a phenotype was calculated for each cell using the mean activation of a subset of relevant species: contractile {SMMHC, SMA, SM22}, synthetic {Col3a1, Eln, TIMP}, and degradative {LAMP1/2, MMP2, S6, MITF, and β-catenin}. a) As it can be seen, the simulated Baseline cell was primarily contractile-synthetic, with non-zero degradative, as expected of a normal cell performing a mechano-sensing and mechano-regulating function to maintain an extracellular matrix experiencing low turnover. b) A representative *Tsc1* KO cell exhibited a shift towards a degradative phenotype with decreases in the degree of contractile and synthetic phenotypic expression.
(TIF)

S7 Fig Lower branch equilibrium solutions only, for different Hill parameters, *EC*_50_ and *n*, which affect the existence and position of the limit point bifurcation. Higher *EC*_50_ dampens signal transmission and therefore reduces the strength of the positive feedback, leading to a shift of the limit point bifurcation towards higher *PI*3*K*. If the signal is sufficiently damped, the limit point bifurcation is not seen. A shift towards higher *PI*3*K* is also seen for increasing *n*, although the effect is not as extreme.
(TIF)

S1 Text Table A. Detailed list of species (nodes) and reactions (edges) for the smooth muscle cell mTOR network structure, with associated references (>100) that motivated the network structure. Inhibition (`NOT’) is denoted by `!’, `AND’ statements are denoted by `&’, and `OR’ statements are constructed by collating all statements with an identical right-hand side.
(DOCX)
