# Supplementary figures and images for: Roles of mTOR in thoracic aortopathy understood by complex intracellular signaling interactions
Source: PLoS Comput Biol. 2021 Dec 13;17(12):e1009683. doi: 10.1371/journal.pcbi.1009683 (PMC8700007; doi:10.1371/journal.pcbi.1009683)

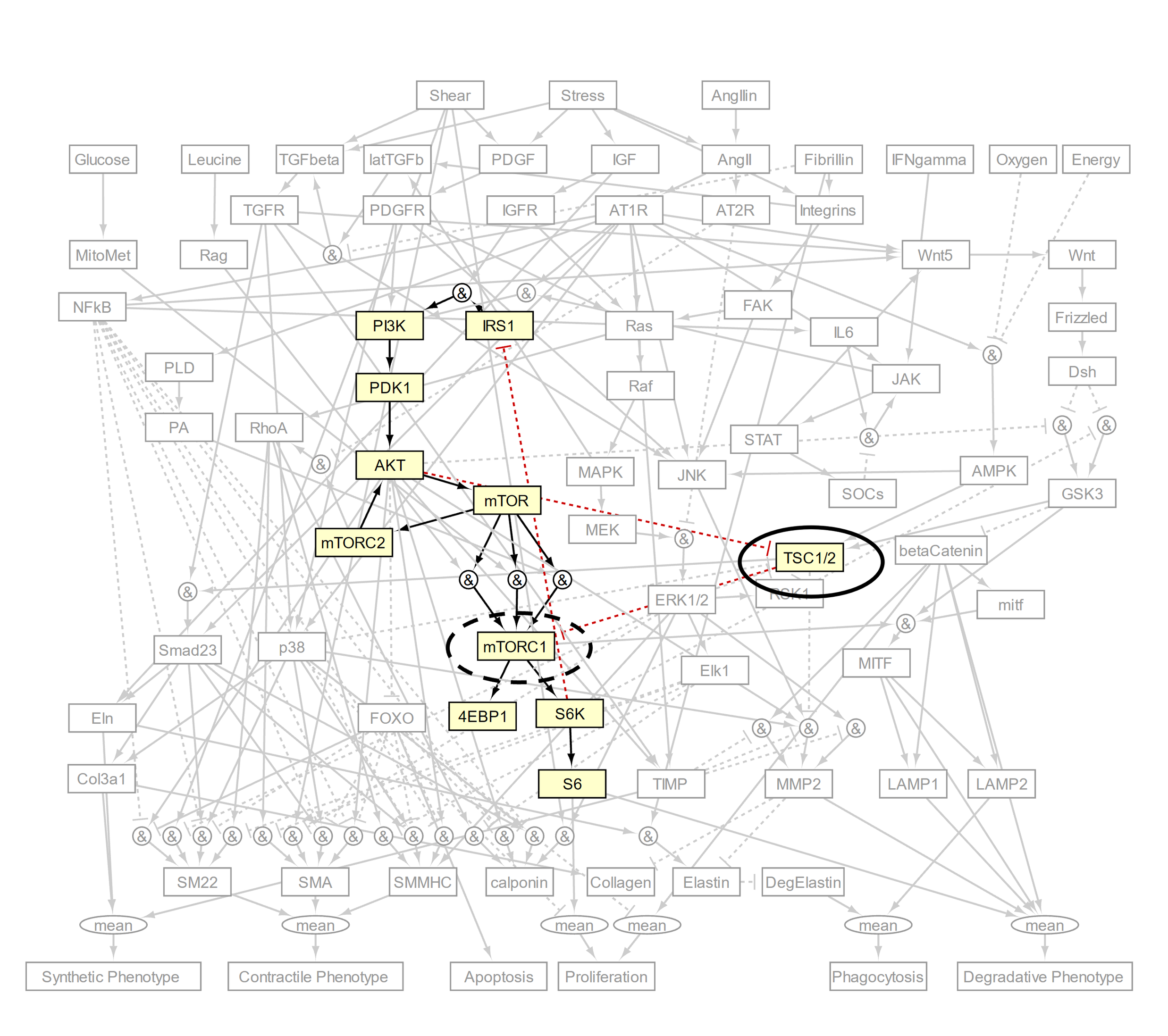

Supplement: S1 Fig — The solid ellipse highlights the TSC1/2 node, which we used to simulate Tsc1 knock-out by setting its ymax = 0. The dashed ellipse highlights the mTORC1 node, which is the primary target of the inhibitor rapamycin. We simulated full inhibition by rapamycin by setting ymax = 0 for the mTORC1 node. See Table A in S1 Text and the associated 105 references that were used to build the overall network. A solid black line indicates activation; a red dashed line indicates inhibition. (TIF) [file pcbi.1009683.s001.tif]

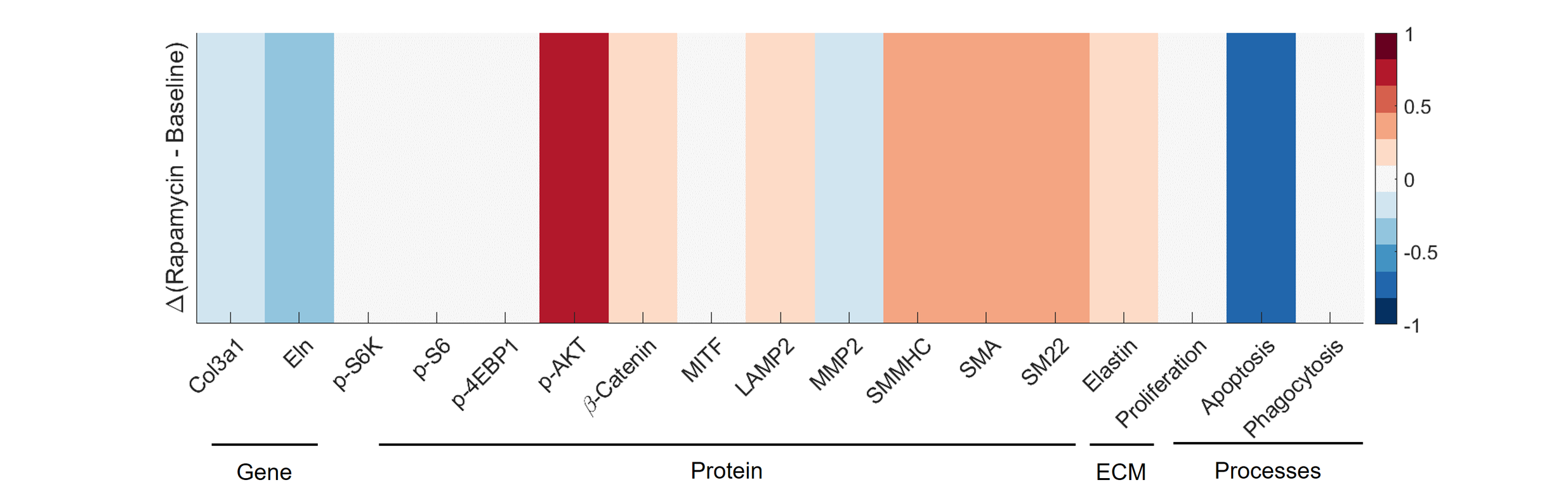

Supplement: S2 Fig — While the experimental data from Li et al. (2020) did not include this comparison, we have added it for completeness. Rapamycin leads to greater activation of p-AKT and contractile signaling, as well as reductions in apoptosis, matrix transcripts, and MMP2 compared to the baseline model. (TIF) [file pcbi.1009683.s002.tif]

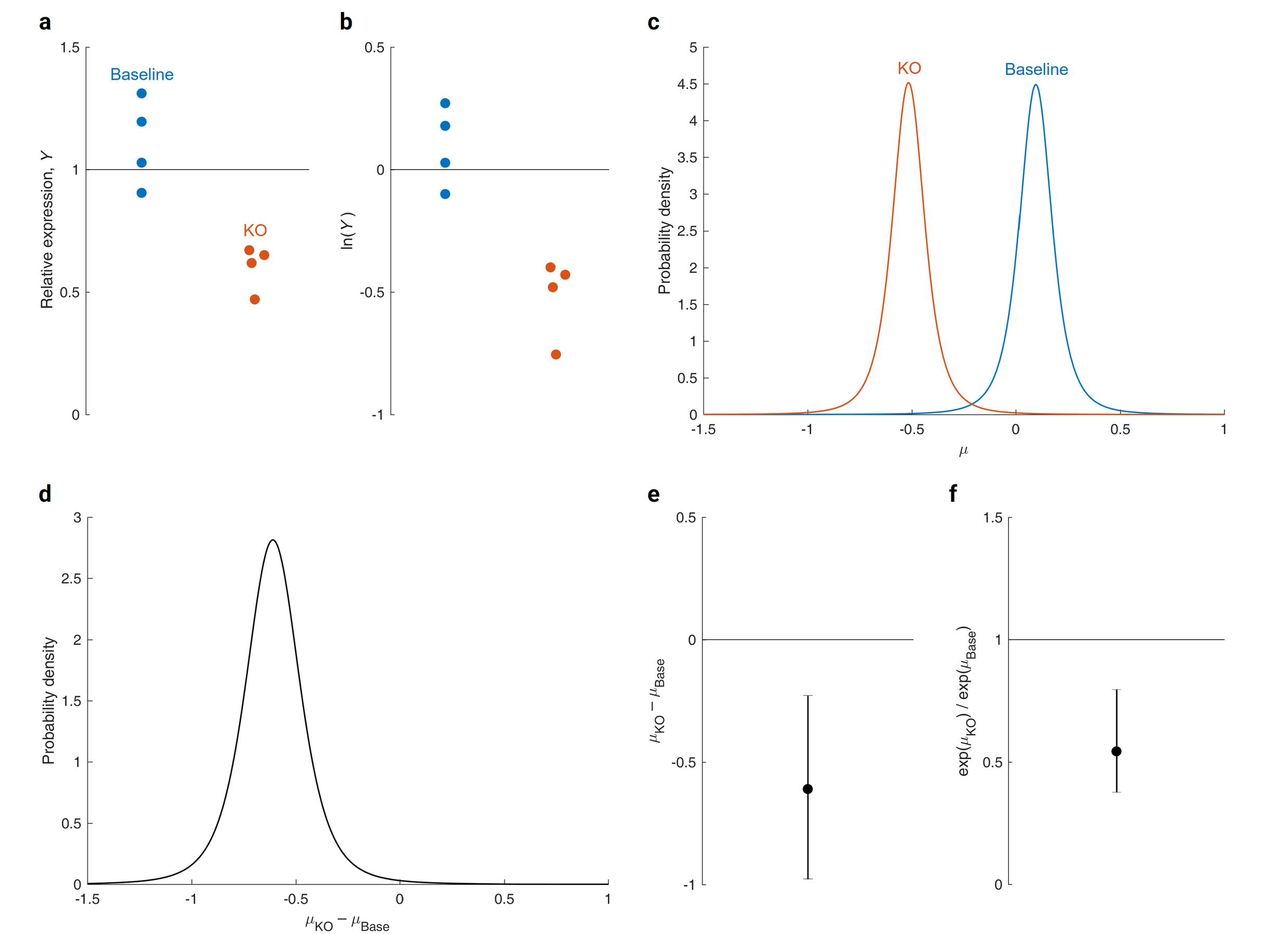

Supplement: S3 Fig — a) Relative expression data for a hypothetical species derived from western blot densitometry normalized to a loading control, with baseline (Base) and Tsc1 null (KO) groups each having a sample size of 4. b) Log-transformation of the relative expression data in (a). c) Posterior distributions for the group-specific median log-expressions μBase and μKO, assuming the log-expression data within each group are normally distributed (i.e., assuming the untransformed data are lognormally distributed). d) Posterior distribution for the difference in median log-expressions, μKO−μBase. e) Point estimate and 95% equi-tailed credible interval for the difference in median log-expressions. f) Point estimate and 95% equi-tailed credible interval for the KO/Baseline ratio of median expressions. The point estimate and interval results shown for all species in Fig 3 correspond to those shown in (f), computed via the methodology presented in Methods. (TIF) [file pcbi.1009683.s003.tif]

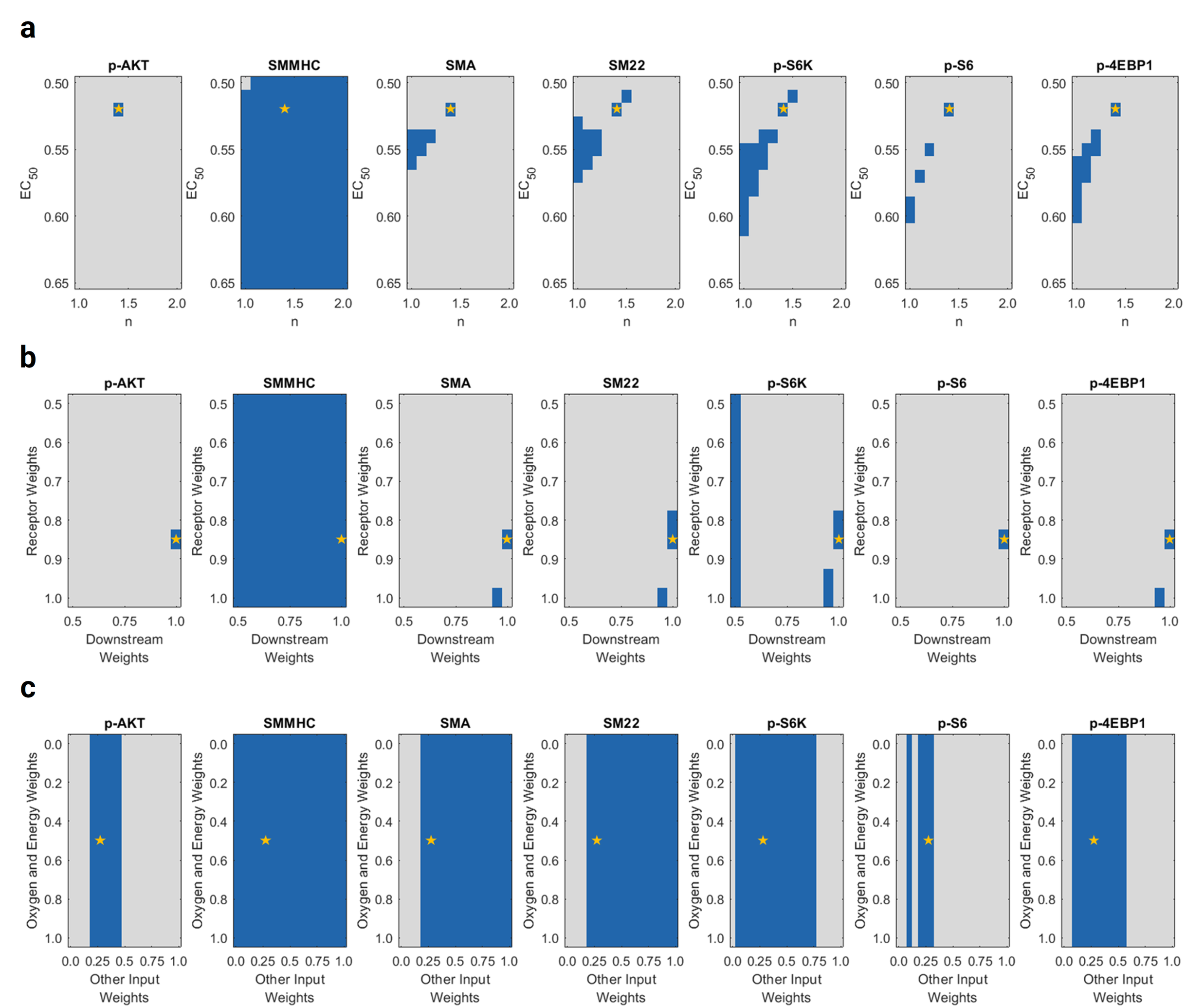

Supplement: S4 Fig — Blue regions indicate combinations where the solution falls within a 95% credible interval of data for the species of interest. The star corresponds to the parameters used in the network model. Combinations of parameters: a) EC50 and n; b) receptor reaction weights and downstream reaction weights; and c) the weight of input oxygen and cellular energy and weight of the remaining model inputs (Glucose, Leucine, Fibrillin). (TIF) [file pcbi.1009683.s004.tif]

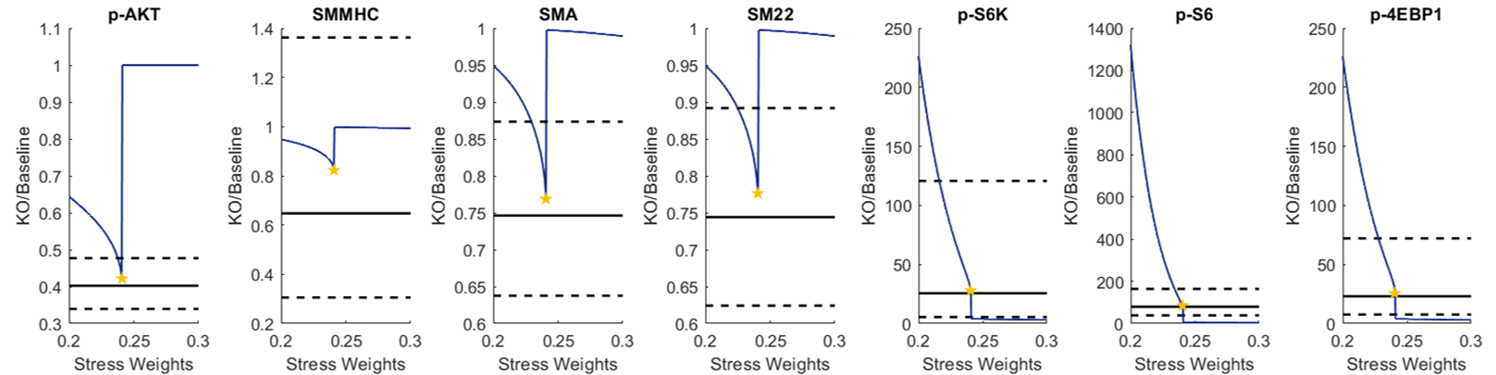

Supplement: S5 Fig — The pressure-induced intramural stress and wall shear stress are kept equal in our simulations. In each panel, the blue line corresponds to the Tsc1 KO/baseline ratio for each species, with the solid and dashed black lines showing the point estimate and 95% credible interval for the KO/baseline ratio of median expressions, based on the experimental data. The star indicates the stress parameters used in the network model. (TIF) [file pcbi.1009683.s005.tif]

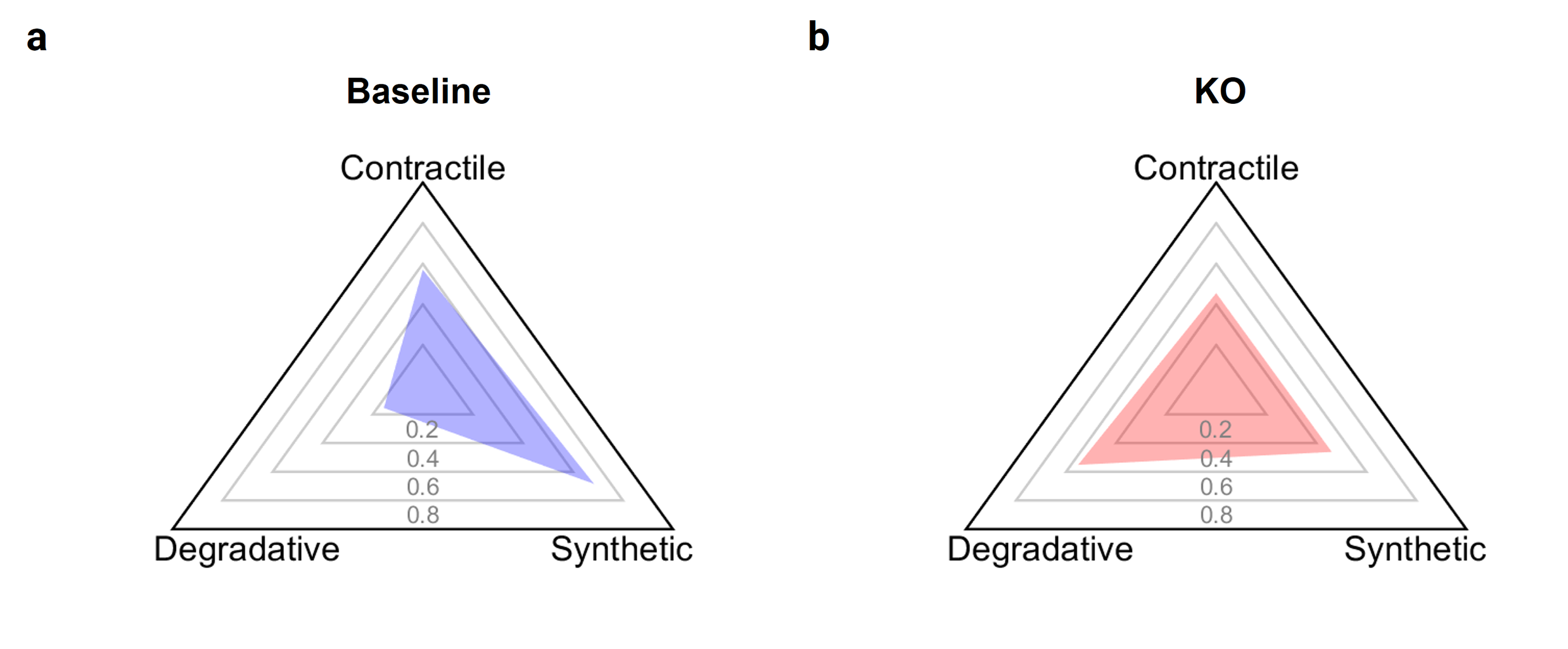

Supplement: S6 Fig — The degree of a phenotype was calculated for each cell using the mean activation of a subset of relevant species: contractile {SMMHC, SMA, SM22}, synthetic {Col3a1, Eln, TIMP}, and degradative {LAMP1/2, MMP2, S6, MITF, and β-catenin}. a) As it can be seen, the simulated Baseline cell was primarily contractile-synthetic, with non-zero degradative, as expected of a normal cell performing a mechano-sensing and mechano-regulating function to maintain an extracellular matrix experiencing low turnover. b) A representative Tsc1 KO cell exhibited a shift towards a degradative phenotype with decreases in the degree of contractile and synthetic phenotypic expression. (TIF) [file pcbi.1009683.s006.tif]

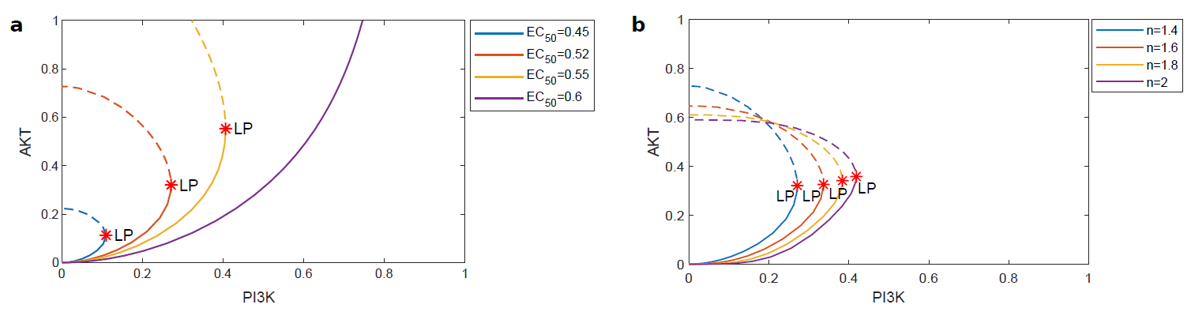

Supplement: S7 Fig — Higher EC50 dampens signal transmission and therefore reduces the strength of the positive feedback, leading to a shift of the limit point bifurcation towards higher PI3K. If the signal is sufficiently damped, the limit point bifurcation is not seen. A shift towards higher PI3K is also seen for increasing n, although the effect is not as extreme. (TIF) [file pcbi.1009683.s007.tif]
