# Supplementary material for: Roles of mTOR in thoracic aortopathy understood by complex intracellular signaling interactions
Source: PLoS Comput Biol. 2021 Dec 13;17(12):e1009683. doi: 10.1371/journal.pcbi.1009683 (PMC8700007; doi:10.1371/journal.pcbi.1009683)
Supplement: S1 Text — Inhibition (`NOT’) is denoted by `!’, `AND’ statements are denoted by `&’, and `OR’ statements are constructed by collating all statements with an identical right-hand side. (DOCX) [file pcbi.1009683.s008.docx]

**Supporting Information**

Roles of mTOR in Thoracic Aortopathy Understood by

Complex Intracellular Signaling Interactions

Ana C. Estrada^1^, Linda Irons^1^, Bruno V. Rego^1^, Guangxin Li^2^, George Tellides^2,3^, Jay D. Humphrey^1,3,*^

^1^Department of Biomedical Engineering, Yale University; New Haven, Connecticut, USA

^2^Department of Surgery, Yale School of Medicine; New Haven, Connecticut, USA

^3^Vascular Biology and Therapeutics Program, Yale School of Medicine; New Haven, Connecticut, USA

*jay.humphrey@yale.edu

**Supporting tables**

**Table A: Detailed list of species (nodes) and reactions (edges) for the smooth muscle cell mTOR network structure, with associated references that motivated the network structure.** Inhibition (`NOT’) is denoted by `!’, `AND’ statements are denoted by `&’, and `OR’ statements are constructed by collating all statements with an identical right-hand side.

| **Edges** | **References** |
| --- | --- |
| => AngIIin | [1,2] |
| => Energy | [3,4] |
| => Fibrillin | [5] |
| => Glucose | [4,6] |
| => IFNgamma | [7] |
| => Leucine | [4,8] |
| => Oxygen | [4,9] |
| => Shear | [10,11] |
| => Stress | [10,12–16] |
| !Akt & !Dsh => GSK3 | [17,18] |
| !Akt => FOXO | [18,19] |
| !Akt => TSC12 | [20,21] |
| !Dsh & !S6K => GSK3 | [17,18] |
| !Elastin => DegElastin | [22,23] |
| !Elk1 & RhoA => calponin | [24,25] |
| !Elk1 & RhoA => SM22 | [24,25] |
| !Elk1 & RhoA => SMA | [24,25] |
| !Elk1 & RhoA => SMMHC | [24,25] |
| !Energy & !Oxygen & AT1R => AMPK | [26] |
| !ERK12 & Shear => SM22 | [27] |
| !ERK12 & Shear => SMA | [27] |
| !ERK12 => TSC12 | [28] |
| !GSK3 => betaCatenin | [17] |
| !MMP2 => Collagen | [22,29] |
| !MMP2 => Elastin | [22,23] |
| !p38 => TSC12 | [30] |
| !RSK1 => TSC12 | [31] |
| !S6K => IRS1 | [19,32] |
| !TSC12 => mTORC1 | [19,26] |
| Akt & !FOXO & !NFkB => calponin | [32–36] |
| Akt & !FOXO & !NFkB => SM22 | [32–36] |
| Akt & !FOXO & !NFkB => SMA | [32–36] |
| Akt & !FOXO & !NFkB => SMMHC | [32,33,35,36] |
| Akt & JNK & p38 & !TIMP & !TSC12 => MMP2 | [15,37,38] |
| Akt => mTOR | [33] |
| Akt => TIMP | [39] |
| AMPK => JNK | [40] |
| AMPK => TSC12 | [26] |
| AngII => AT1R | [2] |
| AngII => AT2R | [2] |
| AngIIin => AngII | [1,2] |
| AT1R & JAK => PI3K | [41–43] |
| AT1R => JAK | [7,44] |
| AT1R => JNK | [45] |
| AT1R => NFkB | [46] |
| AT1R => p38 | [47] |
| AT1R => PLD | [44,48] |
| AT1R => Ras | [49] |
| AT1R => Smad23 | [50] |
| AT1R => TIMP | [51] |
| AT1R => Wnt5 | [52] |
| betaCatenin & !p38 => Proliferation | [53,54] |
| betaCatenin & !TIMP => MMP2 | [53] |
| betaCatenin => LAMP1 | [55,56] |
| betaCatenin => LAMP2 | [55,56] |
| betaCatenin => mitf | [57] |
| Col3a1 => Collagen | [58] |
| DegElastin & LAMP2 => Phagocytosis | [59] |
| Eln & Col3a1 & TIMP => Synthetic | [59] |
| Eln & Fibrillin => Elastin | [60] |
| ERK12 & !TIMP => MMP2 | [37,38] |
| ERK12 & Shear => calponin | [27] |
| ERK12 => Elk1 | [61] |
| ERK12 => RSK1 | [62] |
| ERK12 => SMMHC | [27,61] |
| FAK => JNK | [63] |
| FAK => Ras | [64] |
| Fibrillin => Integrins | [5] |
| FOXO => Apoptosis | [19] |
| Frizzled => Dsh | [17] |
| Glucose => MitoMet | [3] |
| GSK3 => TSC12 | [26] |
| IGF => Eln | [65] |
| IGF => IGFR | [32] |
| IGFR => Ras | [66] |
| IL6 & !SOCs => JAK | [67,68] |
| IFNgamma => JAK | [7] |
| Integrins => FAK | [69] |
| Integrins => latTGFb | [70] |
| IRS1 & IGFR => PI3K | [19,32] |
| JAK => STAT | [7] |
| latTGFb & !Fibrillin => TGFbeta | [71] |
| Leucine => Rag | [8] |
| MAPK => latTGFb | [72,73] |
| MAPK => MEK | [74] |
| MEK & !AT2R => ERK12 | [74,75] |
| mitf & GSK3 & mTORC1 => MITF | [76] |
| MITF => LAMP1 | [77,78] |
| MITF => LAMP2 | [78,79] |
| MitoMet & mTOR => mTORC1 | [3] |
| mTOR => mTORC2 | [80] |
| mTORC1 => p4EBP1 | [81] |
| mTORC1 => S6K | [81] |
| mTORC2 => Akt | [80,82] |
| mTORC2 & !AT2R => RhoA | [83,84] |
| NFkB => IL6 | [85] |
| NFkB => Wnt5 | [86] |
| p38 => Col3a1 | [87] |
| PA & mTOR => mTORC1 | [88,89] |
| PDGF => PDGFR | [15] |
| PDGFR => JNK | [16,37] |
| PDGFR => p38 | [90] |
| PDGFR => Ras | [91,92] |
| PDGFR=> PI3K | [15] |
| PDK1 => Akt | [42] |
| PI3K => PDK1 | [42] |
| PLD => PA | [88] |
| Raf => MAPK | [74] |
| Rag & mTOR => mTORC1 | [8,93] |
| Ras => Raf | [74] |
| Ras => RhoA | [94] |
| S6 & !p38 => Proliferation | [33,54] |
| S6 & MMP2 & LAMP1 & MITF & betaCatenin => Degradative | [59] |
| S6K => S6 | [18,19] |
| Shear => PDGF | [95] |
| Shear => TGFbeta | [11] |
| SMA & SMMHC & SM22=> Contractile | [32,33,59] |
| Smad23 & p38 & !FOXO & !NFkB => SMA | [35,36,96,97] |
| Smad23 & p38 & !FOXO & !NFkB => calponin | [35,36,96,97] |
| Smad23 & p38 & !FOXO & !NFkB => SM22 | [35,36,96,97] |
| Smad23 & p38 & !FOXO & !NFkB => SMMHC | [35,36,97] |
| Smad23 => Col3a1 | [98,99] |
| Smad23 => Eln | [100] |
| STAT => SOCs | [68,101] |
| STAT => Wnt5 | [86] |
| Stress => AngII | [12] |
| Stress => IGF | [13] |
| Stress => Integrins | [14] |
| Stress => PDGF | [15,16] |
| Stress => TGFbeta | [12] |
| TGFbeta => TGFR | [12] |
| TGFR & TSC12 => Smad23 | [102–104] |
| TGFR => p38 | [104] |
| TGFR => TIMP | [105] |
| TGFR => Wnt5 | [86] |
| TGFR=> JNK | [104] |
| Wnt => Frizzled | [17] |
| Wnt5 => Wnt | [86] |

**References**

1. Griendling KK, Ushio-Fukai M, Lassègue B, Alexander RW. Angiotensin II Signaling in Vascular Smooth Muscle. Hypertension [Internet]. 1997 Jan 1;29(1 II):366–73. Available from: https://www.ahajournals.org/doi/abs/10.1161/01.hyp.29.1.366

2. Hafizi S, Wang X, Chester AH, Yacoub MH, Proud CG. ANG II activates effectors of mTOR via PI3-K signaling in human coronary smooth muscle cells. Am J Physiol Circ Physiol [Internet]. 2004 Sep;287(3):H1232–8. Available from: https://www.physiology.org/doi/10.1152/ajpheart.00040.2004

3. Dennis PB, Jaeschke A, Saitoh M, Fowler B, Kozma SC, Thomas G. Mammalian TOR: A Homeostatic ATP Sensor. Science (80- ) [Internet]. 2001 Nov 2;294(5544):1102–5. Available from: https://science.sciencemag.org/content/294/5544/1102

4. Gitto SB, Altomare DA. Recent insights into the pathophysiology of mTOR pathway dysregulation. Res Rep Biol [Internet]. 2015 Jan 29;6:1–16. Available from: https://www.dovepress.com/recent-insights-into-the-pathophysiology-of-mtor-pathway-dysregulation-peer-reviewed-fulltext-article-RRB

5. Bax DV, Bernard SE, Lomas A, Morgan A, Humphries J, Shuttleworth CA, et al. Cell Adhesion to Fibrillin-1 Molecules and Microfibrils Is Mediated by α5β1 and αvβ3 Integrins *. J Biol Chem [Internet]. 2003 Sep 5;278(36):34605–16. Available from: http://www.jbc.org/article/S0021925820837818/fulltext

6. Inoki K, Zhu T, Guan K-L. TSC2 mediates cellular energy response to control cell growth and survival. Cell [Internet]. 2003 Nov 26;115(5):577–90. Available from: https://pubmed.ncbi.nlm.nih.gov/14651849/

7. Marrero MB, Schieffer B, Paxton WG, Heerdt L, Berk BC, Delafontaine P, et al. Direct stimulation of Jak/STAT pathway by the angiotensin II AT1 receptor. Nat 1995 3756528 [Internet]. 1995 May 18;375(6528):247–50. Available from: https://www.nature.com/articles/375247a0

8. Kim JH, Lee C, Lee M, Wang H, Kim K, Park SJ, et al. Control of leucine-dependent mTORC1 pathway through chemical intervention of leucyl-tRNA synthetase and RagD interaction. Nat Commun 2017 81 [Internet]. 2017 Sep 29;8(1):1–15. Available from: https://www.nature.com/articles/s41467-017-00785-0

9. Brugarolas J, Lei K, Hurley RL, Manning BD, Reiling JH, Hafen E, et al. Regulation of mTOR function in response to hypoxia by REDD1 and the TSC1/TSC2 tumor suppressor complex. Genes Dev [Internet]. 2004 Dec 1;18(23):2893–904. Available from: http://genesdev.cshlp.org/content/18/23/2893.full

10. Humphrey JD. Vascular Adaptation and Mechanical Homeostasis at Tissue, Cellular, and Sub-cellular Levels. Cell Biochem Biophys 2007 502 [Internet]. 2007 Oct 24;50(2):53–78. Available from: https://link.springer.com/article/10.1007/s12013-007-9002-3

11. Ueba H, Kawakami M, Yaginuma T. Shear Stress as an Inhibitor of Vascular Smooth Muscle Cell Proliferation. Arterioscler Thromb Vasc Biol [Internet]. 1997;17(8):1512–6. Available from: https://www.ahajournals.org/doi/abs/10.1161/01.atv.17.8.1512

12. Li Q, Muragaki Y, Hatamura I, Ueno H, Ooshima A. Stretch-induced collagen synthesis in cultured smooth muscle cells from rabbit aortic media and a possible involvement of angiotensin II and transforming growth factor-β. J Vasc Res. 1998 Mar;35(2):93–103.

13. Cheng J, Du J. Mechanical Stretch Simulates Proliferation of Venous Smooth Muscle Cells Through Activation of the Insulin-Like Growth Factor-1 Receptor. Arterioscler Thromb Vasc Biol [Internet]. 2007 Aug 1;27(8):1744–51. Available from: https://www.ahajournals.org/doi/abs/10.1161/atvbaha.107.147371

14. Goldschmidt ME, McLeod KJ, Taylor WR. Integrin-Mediated Mechanotransduction in Vascular Smooth Muscle Cells. Circ Res [Internet]. 2001 Apr 13;88(7):674–80. Available from: https://www.ahajournals.org/doi/abs/10.1161/hh0701.089749

15. Seo KW, Lee SJ, Kim YH, Bae JU, Park SY, Bae SS, et al. Mechanical Stretch Increases MMP-2 Production in Vascular Smooth Muscle Cells via Activation of PDGFR-β/Akt Signaling Pathway. PLoS One. 2013 Aug 7;8(8).

16. Hu Y, Böck G, Wick G, Xu Q. Activation of PDGF receptor α in vascular smooth muscle cells by mechanical stress. FASEB J [Internet]. 1998 Sep 1;12(12):1135–42. Available from: https://faseb.onlinelibrary.wiley.com/doi/full/10.1096/fasebj.12.12.1135

17. Dominguez I, Green JBA. Missing Links in GSK3 Regulation. Dev Biol. 2001 Jul 15;235(2):303–13.

18. Zhang HH, Lipovsky AI, Dibble CC, Sahin M, Manning BD. S6K1 regulates GSK3 under conditions of mTOR-dependent feedback inhibition of Akt. Mol Cell [Internet]. 2006 Oct 20;24(2):185. Available from: /pmc/articles/PMC1880887/

19. Shah OJ, Wang Z, Hunter T. Inappropriate Activation of the TSC/Rheb/mTOR/S6K Cassette Induces IRS1/2 Depletion, Insulin Resistance, and Cell Survival Deficiencies. Curr Biol. 2004 Sep 21;14(18):1650–6.

20. Inoki K, Li Y, Zhu T, Wu J, Guan K-L. TSC2 is phosphorylated and inhibited by Akt and suppresses mTOR signalling. Nat Cell Biol 2002 49 [Internet]. 2002 Aug 12;4(9):648–57. Available from: https://www.nature.com/articles/ncb839

21. Manning BD, Tee AR, Logsdon MN, Blenis J, Cantley LC. Identification of the Tuberous Sclerosis Complex-2 Tumor Suppressor Gene Product Tuberin as a Target of the Phosphoinositide 3-Kinase/Akt Pathway. Mol Cell. 2002 Jul 1;10(1):151–62.

22. Longo GM, Xiong W, Greiner TC, Zhao Y, Fiotti N, Baxter BT. Matrix metalloproteinases 2 and 9 work in concert to produce aortic aneurysms. J Clin Invest. 2002 Sep 1;110(5):625–32.

23. Freestone T, Turner RJ, Coady A, Higman DJ, Greenhalgh RM, Powell JT. Inflammation and Matrix Metalloproteinases in the Enlarging Abdominal Aortic Aneurysm. Arterioscler Thromb Vasc Biol [Internet]. 1995;15(8):1145–51. Available from: https://www.ahajournals.org/doi/abs/10.1161/01.ATV.15.8.1145

24. Wang Z, Wang D-Z, Hockemeyer D, McAnally J, Nordheim A, Olson EN. Myocardin and ternary complex factors compete for SRF to control smooth muscle gene expression. Nat 2004 4286979 [Internet]. 2004 Mar 11;428(6979):185–9. Available from: https://www.nature.com/articles/nature02382

25. Mack CP, Somlyo A V., Hautmann M, Somlyo AP, Owens GK. Smooth Muscle Differentiation Marker Gene Expression Is Regulated by RhoA-mediated Actin Polymerization *. J Biol Chem [Internet]. 2001 Jan 5;276(1):341–7. Available from: http://www.jbc.org/article/S0021925818442445/fulltext

26. Inoki K, Ouyang H, Zhu T, Lindvall C, Wang Y, Zhang X, et al. TSC2 Integrates Wnt and Energy Signals via a Coordinated Phosphorylation by AMPK and GSK3 to Regulate Cell Growth. Cell. 2006 Sep 8;126(5):955–68.

27. Shi Z-D, Abraham G, Tarbell JM. Shear Stress Modulation of Smooth Muscle Cell Marker Genes in 2-D and 3-D Depends on Mechanotransduction by Heparan Sulfate Proteoglycans and ERK1/2. Agarwal S, editor. PLoS One [Internet]. 2010 Aug 16;5(8):e12196. Available from: http://dx.plos.org/10.1371/journal.pone.0012196

28. Ma L, Chen Z, Erdjument-Bromage H, Tempst P, Pandolfi PP. Phosphorylation and Functional Inactivation of TSC2 by Erk: Implications for Tuberous Sclerosisand Cancer Pathogenesis. Cell. 2005 Apr 22;121(2):179–93.

29. Nagase H, Visse R, Murphy G. Structure and function of matrix metalloproteinases and TIMPs. Cardiovasc Res [Internet]. 2006 Feb 15;69(3):562–73. Available from: https://academic.oup.com/cardiovascres/article/69/3/562/272258

30. Li Y, Inoki K, Vacratsis P, Guan KL. The p38 and MK2 kinase cascade phosphorylates tuberin, the tuberous sclerosis 2 gene product, and enhances its interaction with 14-3-3. J Biol Chem. 2003 Apr 18;278(16):13663–71.

31. Roux PP, Ballif BA, Anjum R, Gygi SP, Blenis J. Tumor-promoting phorbol esters and activated Ras inactivate the tuberous sclerosis tumor suppressor complex via p90 ribosomal S6 kinase. Proc Natl Acad Sci U S A. 2004 Sep 14;101(37):13489–94.

32. Martin KA, Merenick BL, Ding M, Fetalvero KM, Rzucidlo EM, Kozul CD, et al. Rapamycin promotes vascular smooth muscle cell differentiation through insulin receptor substrate-1/phosphatidylinositol 3-kinase/Akt2 feedback signaling. J Biol Chem. 2007 Dec 7;282(49):36112–20.

33. Hegner B, Lange M, Kusch A, Essin K, Sezer O, Schulze-Lohoff E, et al. mTOR Regulates Vascular Smooth Muscle Cell Differentiation From Human Bone Marrow–Derived Mesenchymal Progenitors. Arterioscler Thromb Vasc Biol [Internet]. 2009 Feb 1;29(2):232–8. Available from: https://www.ahajournals.org/doi/abs/10.1161/atvbaha.108.179457

34. Yun SJ, Ha JM, Kim EK, Kim YW, Jin SY, Lee DH, et al. Akt1 isoform modulates phenotypic conversion of vascular smooth muscle cells. Biochim Biophys Acta - Mol Basis Dis. 2014 Nov 1;1842(11):2184–92.

35. Liu Z-P, Wang Z, Yanagisawa H, Olson EN. Phenotypic Modulation of Smooth Muscle Cells through Interaction of Foxo4 and Myocardin. Dev Cell [Internet]. 2005 Aug 1;9(2):261–70. Available from: http://www.cell.com/article/S1534580705002133/fulltext

36. Tang R, Zheng X-L, Callis TE, Stansfield WE, He J, Baldwin AS, et al. Myocardin inhibits cellular proliferation by inhibiting NF-κB(p65)-dependent cell cycle progression. Proc Natl Acad Sci [Internet]. 2008 Mar 4;105(9):3362–7. Available from: https://www.pnas.org/content/105/9/3362

37. Risinger GM, Hunt TS, Updike DL, Bullen EC, Howard EW. Matrix metalloproteinase-2 expression by vascular smooth muscle cells is mediated by both stimulatory and inhibitory signals in response to growth factors. J Biol Chem. 2006 Sep 8;281(36):25915–25.

38. Cui Y, Sun Y-W, Lin H-S, Su W-M, Fang Y, Zhao Y, et al. Platelet-derived growth factor-BB induces matrix metalloproteinase-2 expression and rat vascular smooth muscle cell migration via ROCK and ERK/p38 MAPK pathways. Mol Cell Biochem [Internet]. 2014;393(1–2):255–63. Available from: https://pubmed.ncbi.nlm.nih.gov/24792035/

39. Yu H, Fellows A, Foote K, Yang Z, Figg N, Littlewood T, et al. FOXO3a (Forkhead Transcription Factor O Subfamily Member 3a) Links Vascular Smooth Muscle Cell Apoptosis, Matrix Breakdown, Atherosclerosis, and Vascular Remodeling Through a Novel Pathway Involving MMP13 (Matrix Metalloproteinase 13). Arterioscler Thromb Vasc Biol [Internet]. 2018;38(3):555–65. Available from: https://www.ahajournals.org/doi/abs/10.1161/ATVBAHA.117.310502

40. Yun H, Kim H-S, Lee S, Kang I, Kim SS, Choe W, et al. AMP kinase signaling determines whether c-Jun N-terminal kinase promotes survival or apoptosis during glucose deprivation. Carcinogenesis [Internet]. 2009;30(3):529–37. Available from: https://pubmed.ncbi.nlm.nih.gov/19037093/

41. Saward L, Zahradka P. Angiotensin II activates phosphatidylinositol 3-kinase in vascular smooth muscle cells. Circ Res [Internet]. 1997;81(2):249–57. Available from: https://pubmed.ncbi.nlm.nih.gov/9242186/

42. Dugourd C, Gervais M, Corvol P, Monnot C. Akt is a major downstream target of PI3-kinase involved in angiotensin II-induced proliferation. Hypertens (Dallas, Tex 1979) [Internet]. 2003 Apr 1;41(4):882–90. Available from: http://www.ncbi.nlm.nih.gov/pubmed/12623864

43. Yamada O, Ozaki K, Akiyama M, Kawauchi K. JAK–STAT and JAK–PI3K–mTORC1 Pathways Regulate Telomerase Transcriptionally and Posttranslationally in ATL Cells. Mol Cancer Ther [Internet]. 2012 May 1;11(5):1112–21. Available from: https://mct.aacrjournals.org/content/11/5/1112

44. Yu C, Jeremy RW. Angiotensin, transforming growth factor β and aortic dilatation in Marfan syndrome: Of mice and humans. IJC Hear Vasc. 2018 Mar 1;18:71–80.

45. Schmitz U, Ishida T, Ishida M, Surapisitchat J, Hasham MI, Pelech S, et al. Angiotensin II Stimulates p21-Activated Kinase in Vascular Smooth Muscle Cells. Circ Res [Internet]. 1998 Jun 29;82(12):1272–8. Available from: https://www.ahajournals.org/doi/abs/10.1161/01.res.82.12.1272

46. Ruiz-Ortega M, Lorenzo O, Rupérez M, König S, Wittig B, Egido J. Angiotensin II Activates Nuclear Transcription Factor κB Through AT1 and AT2 in Vascular Smooth Muscle Cells. Circ Res [Internet]. 2000 Jun 23;86(12):1266–72. Available from: https://www.ahajournals.org/doi/abs/10.1161/01.res.86.12.1266

47. Subramanian V, Golledge J, Heywood EB, Bruemmer D, Daugherty A. Regulation of PPARγ by Angiotensin II via TGF-β1 Activated p38 MAP Kinase in Aortic Smooth Muscle Cells. Arterioscler Thromb Vasc Biol [Internet]. 2012 Feb;32(2):397. Available from: /pmc/articles/PMC3262055/

48. Lassègue B, Alexander RW, Clark M, Akers M, Griendling KK. Phosphatidylcholine is a major source of phosphatidic acid and diacylglycerol in angiotensin II-stimulated vascular smooth-muscle cells. Biochem J [Internet]. 1993 Jun 1;292(2):509–17. Available from: /biochemj/article/292/2/509/30209/Phosphatidylcholine-is-a-major-source-of

49. Eguchi S, Matsumoto T, Motley ED, Utsunomiya H, Inagami T. Identification of an Essential Signaling Cascade for Mitogen-activated Protein Kinase Activation by Angiotensin II in Cultured Rat Vascular Smooth Muscle Cells: POSSIBLE REQUIREMENT OF Gq-MEDIATED p21ras ACTIVATION COUPLED TO A Ca2+/CALMODULIN-SENSITIVE TYROSINE KINASE *. J Biol Chem [Internet]. 1996 Jun 14;271(24):14169–75. Available from: http://www.jbc.org/article/S0021925818467816/fulltext

50. Kuang SQ, Geng L, Prakash SK, Cao JM, Guo S, Villamizar C, et al. Aortic remodeling after transverse aortic constriction in mice is attenuated with AT1 receptor blockade. Arterioscler Thromb Vasc Biol [Internet]. 2013 Sep;33(9):2172–9. Available from: http://www.ncbi.nlm.nih.gov/pubmed/23868934

51. Castoldi G, Di Gioia CRT, Pieruzzi F, D’Orlando C, Van De Greef WMM, Busca G, et al. ANG II increases TIMP-1 expression in rat aortic smooth muscle cells in vivo. Am J Physiol Heart Circ Physiol [Internet]. 2003 Feb 1;284(2). Available from: https://pubmed.ncbi.nlm.nih.gov/12388255/

52. Zhao Y, Wang C, Wang C, Hong X, Miao J, Liao Y, et al. An essential role for Wnt/β-catenin signaling in mediating hypertensive heart disease. Sci Reports 2018 81 [Internet]. 2018 Jun 12;8(1):1–14. Available from: https://www.nature.com/articles/s41598-018-27064-2

53. Riascos-Bernal DF, Chinnasamy P, Gross JN, Almonte V, Egaña-Gorroño L, Parikh D, et al. Inhibition of smooth muscle β-catenin hinders neointima formation after vascular injury. Arterioscler Thromb Vasc Biol [Internet]. 2017 May 1;37(5):879. Available from: /pmc/articles/PMC5408313/

54. Wu Y, Zhou J, Wang H, Wu Y, Gao Q, Wang L, et al. The activation of p38 MAPK limits the abnormal proliferation of vascular smooth muscle cells induced by high sodium concentrations. Int J Mol Med [Internet]. 2016 Jan 1;37(1):74–82. Available from: http://www.spandidos-publications.com/10.3892/ijmm.2015.2394/abstract

55. Tejeda-Muñoz N, Albrecht L V., Bui MH, Robertis EM De. Wnt canonical pathway activates macropinocytosis and lysosomal degradation of extracellular proteins. Proc Natl Acad Sci [Internet]. 2019 May 21;116(21):10402–11. Available from: https://www.pnas.org/content/116/21/10402

56. Albrecht L V, Tejeda-Muñ Oz N, Bui MH, Piccolo S, Christofk HR, De EM, et al. Article GSK3 Inhibits Macropinocytosis and Lysosomal Activity through the Wnt Destruction Complex Machinery GSK3 Inhibits Macropinocytosis and Lysosomal Activity through the Wnt Destruction Complex Machinery. CellReports [Internet]. 2020;32:107973. Available from: https://doi.org/10.1016/j.celrep.2020.107973

57. Cao J, Tyburczy ME, Moss J, Darling TN, Widlund HR, Kwiatkowski DJ. Tuberous sclerosis complex inactivation disrupts melanogenesis via mTORC1 activation. J Clin Invest. 2017 Jan 3;127(1):349–64.

58. Liu X, Wu H, Byrne M, Krane S, Jaenisch R. Type III collagen is crucial for collagen I fibrillogenesis and for normal cardiovascular development. Proc Natl Acad Sci [Internet]. 1997 Mar 4;94(5):1852–6. Available from: https://www.pnas.org/content/94/5/1852

59. Li G, Wang M, Caulk AW, Cilfone NA, Gujja S, Qin L, et al. Chronic mTOR activation induces a degradative smooth muscle cell phenotype. J Clin Invest [Internet]. 2020 Mar 2;130(3):1233–51. Available from: https://pubmed.ncbi.nlm.nih.gov/32039915/

60. Shin SJ, Yanagisawa H. Recent updates on the molecular network of elastic fiber formation. Essays Biochem [Internet]. 2019 Sep 13;63(3):365–76. Available from: /essaysbiochem/article/63/3/365/218806/Recent-updates-on-the-molecular-network-of-elastic

61. Schauwienold D, Plum C, Helbing T, Voigt P, Bobbert T, Hoffmann D, et al. ERK1/2-Dependent Contractile Protein Expression in Vascular Smooth Muscle Cells. Hypertension [Internet]. 2003 Mar 1;41(3 I):546–52. Available from: https://www.ahajournals.org/doi/abs/10.1161/01.HYP.0000054213.37471.84

62. Xu S, Bayat H, Hou X, Jiang B. Ribosomal S6 kinase-1 modulates interleukin-1β-induced persistent activation of NF-κB through phosphorylation of IκBβ. https://doi.org/101152/ajpcell005522005 [Internet]. 2006 Dec;291(6):1336–45. Available from: https://journals.physiology.org/doi/abs/10.1152/ajpcell.00552.2005

63. Sundberg LJ, Galante LM, Bill HM, Mack CP, Taylor JM. An endogenous inhibitor of focal adhesion kinase blocks Rac1/JNK but not Ras/ERK-dependent signaling in vascular smooth muscle cells. J Biol Chem [Internet]. 2003 Aug 8;278(32):29783–91. Available from: https://pubmed.ncbi.nlm.nih.gov/12782622/

64. Schlaepfer DD, Hanks SK, Hunter T, van der Geer P. Integrin-mediated signal transduction linked to Ras pathway by GRB2 binding to focal adhesion kinase. Nature [Internet]. 1994;372(6508):786–91. Available from: https://pubmed.ncbi.nlm.nih.gov/7997267/

65. Wolfe BL, Rich CB, Goud HD, Terpstra AJ, Bashir M, Rosenbloom J, et al. Insulin-like growth factor-I regulates transcription of the elastin gene. J Biol Chem. 1993 Jun 15;268(17):12418–26.

66. Duan C. The chemotactic and mitogenic responses of vascular smooth muscle cells to insulin-like growth factor-I require the activation of ERK1/2. Mol Cell Endocrinol. 2003 Aug 29;206(1–2):75–83.

67. Watanabe S, Mu W, Kahn A, Jing N, Li J, Lan H, et al. Role of JAK/STAT pathway in IL-6-induced activation of vascular smooth muscle cells. Am J Nephrol [Internet]. 2004 Jul 9;24(4):387–92. Available from: https://europepmc.org/article/med/15256805

68. Lee CK, Raz R, Gimeno R, Gertner R, Wistinghausen B, Takeshita K, et al. STAT3 Is a Negative Regulator of Granulopoiesis but Is Not Required for G-CSF-Dependent Differentiation. Immunity. 2002 Jul 1;17(1):63–72.

69. Lehoux S, Esposito B, Merval R, Tedgui A. Differential Regulation of Vascular Focal Adhesion Kinase by Steady Stretch and Pulsatility. Circulation [Internet]. 2005 Feb 8;111(5):643–9. Available from: https://www.ahajournals.org/doi/abs/10.1161/01.cir.0000154548.16191.2f

70. Turner CJ, Badu-Nkansah K, Crowley D, van der Flier A, Hynes RO. α5 and αv integrins cooperate to regulate vascular smooth muscle and neural crest functions in vivo. Development. 2015 Feb 15;142(4):797–808.

71. Zilberberg L, Todorovic V, Dabovic B, Horiguchi M, Couroussé T, Sakai LY, et al. Specificity of latent TGF-β binding protein (LTBP) incorporation into matrix: Role of fibrillins and fibronectin. J Cell Physiol [Internet]. 2012 Dec 1;227(12):3828–36. Available from: https://onlinelibrary.wiley.com/doi/full/10.1002/jcp.24094

72. Chavez RJ, Haney RM, Cuadra RH, Ganguly R, Adapala RK, Thodeti CK, et al. Upregulation of thrombospondin-1 expression by leptin in vascular smooth muscle cells via JAK2- and MAPK-dependent pathways. https://doi.org/101152/ajpcell000082012 [Internet]. 2012 Jul 15;303(2):179–91. Available from: https://journals.physiology.org/doi/abs/10.1152/ajpcell.00008.2012

73. McGillicuddy FC, O’Toole D, Hickey JA, Gallagher WM, Dawson KA, Keenan AK. TGF-β1-induced thrombospondin-1 expression through the p38 MAPK pathway is abolished by fluvastatin in human coronary artery smooth muscle cells. Vascul Pharmacol. 2006 Jun 1;44(6):469–75.

74. Dong L-H, Wen J-K, Liu G, McNutt MA, Miao S-B, Gao R, et al. Blockade of the Ras–Extracellular Signal–Regulated Kinase 1/2 Pathway Is Involved in Smooth Muscle 22α–Mediated Suppression of Vascular Smooth Muscle Cell Proliferation and Neointima Hyperplasia. Arterioscler Thromb Vasc Biol [Internet]. 2010 Apr 1;30(4):683–91. Available from: https://www.ahajournals.org/doi/abs/10.1161/atvbaha.109.200501

75. Habashi JP, Doyle JJ, Holm TM, Aziz H, Schoenhoff F, Bedja D, et al. Angiotensin II Type 2 Receptor Signaling Attenuates Aortic Aneurysm in Mice Through ERK Antagonism. Science (80- ) [Internet]. 2011 Apr 15;332(6027):361–5. Available from: https://science.sciencemag.org/content/332/6027/361

76. Ngeow KC, Friedrichsen HJ, Li L, Zeng Z, Andrews S, Volpon L, et al. BRAF/MAPK and GSK3 signaling converges to control MITF nuclear export. Proc Natl Acad Sci U S A. 2018 Sep 11;115(37):E8668–77.

77. Ploper D, Taelman VF, Robert L, Perez BS, Titz B, Chen H-W, et al. MITF drives endolysosomal biogenesis and potentiates Wnt signaling in melanoma cells. Proc Natl Acad Sci [Internet]. 2015 Feb 3;112(5):E420–9. Available from: https://www.pnas.org/content/112/5/E420

78. Asrani K, Murali S, Lam B, Na C-H, Phatak P, Sood A, et al. mTORC1 feedback to AKT modulates lysosomal biogenesis through MiT/TFE regulation. J Clin Invest [Internet]. 2019 Dec 2;129(12):5584–99. Available from: https://doi.org/10.1172/JCI128287.

79. Lu S, Sung T, Lin N, Abraham RT, Jessen BA. Lysosomal adaptation: How cells respond to lysosomotropic compounds. PLoS One [Internet]. 2017 Mar 1;12(3):e0173771. Available from: https://journals.plos.org/plosone/article?id=10.1371/journal.pone.0173771

80. Schaub T, Gürgen D, Maus D, Lange C, Tarabykin V, Dragun D, et al. mTORC1 and mTORC2 Differentially Regulate Cell Fate Programs to Coordinate Osteoblastic Differentiation in Mesenchymal Stromal Cells. Sci Reports 2019 91 [Internet]. 2019 Dec 27;9(1):1–19. Available from: https://www.nature.com/articles/s41598-019-56237-w

81. Martin KA, Rzucidlo EM, Merenick BL, Fingar DC, Brown DJ, Wagner RJ, et al. The mTOR/p70 S6K1 pathway regulates vascular smooth muscle cell differentiation. Am J Physiol - Cell Physiol. 2004 Mar;286(3 55-3).

82. Krymskaya VP, Snow J, Cesarone G, Khavin I, Goncharov DA, Lim PN, et al. mTOR is required for pulmonary arterial vascular smooth muscle cell proliferation under chronic hypoxia. FASEB J [Internet]. 2011 Jun 1;25(6):1922–33. Available from: https://faseb.onlinelibrary.wiley.com/doi/full/10.1096/fj.10-175018

83. Jacinto E, Loewith R, Schmidt A, Lin S, Rüegg MA, Hall A, et al. Mammalian TOR complex 2 controls the actin cytoskeleton and is rapamycin insensitive. Nat Cell Biol [Internet]. 2004 Nov;6(11):1122–8. Available from: https://pubmed.ncbi.nlm.nih.gov/15467718/

84. Savoia C, Ebrahimian T, He Y, Gratton JP, Schiffrin EL, Touyz RM. Angiotensin II/AT2 receptor-induced vasodilation in stroke-prone spontaneously hypertensive rats involves nitric oxide and cGMP-dependent protein kinase. J Hypertens [Internet]. 2006;24(12):2417–22. Available from: https://pubmed.ncbi.nlm.nih.gov/17082724/

85. Zampetaki A, Zhang Z, Hu Y, Xu Q. Biomechanical stress induces IL-6 expression in smooth muscle cells via Ras/Rac1-p38 MAPK-NF-κB signaling pathways. https://doi.org/101152/ajpheart009192004 [Internet]. 2005 Jun;288(6 57-6):2946–54. Available from: https://journals.physiology.org/doi/abs/10.1152/ajpheart.00919.2004

86. Katoh M, Katoh M. Transcriptional mechanisms of WNT5A based on NF-κB, Hedgehog, TGFβ, and Notch signaling cascades. Int J Mol Med [Internet]. 2009 Jun 1;23(6):763–9. Available from: http://www.spandidos-publications.com/10.3892/ijmm_00000190/abstract

87. Touyz RM, He G, El Mabrouk M, Schiffrin EL. p38 Map kinase regulates vascular smooth muscle cell collagen synthesis by angiotensin II in SHR but not in WKY. Hypertens (Dallas, Tex 1979) [Internet]. 2001;37(2 Pt 2):574–80. Available from: https://pubmed.ncbi.nlm.nih.gov/11230337/

88. Hornberger TA, Chu WK, Mak YW, Hsiung JW, Huang SA, Chien S. The role of phospholipase D and phosphatidic acid in the mechanical activation of mTOR signaling in skeletal muscle. Proc Natl Acad Sci U S A. 2006 Mar 21;103(12):4741–6.

89. You JS, Frey JW, Hornberger TA. Mechanical Stimulation Induces mTOR Signaling via an ERK-Independent Mechanism: Implications for a Direct Activation of mTOR by Phosphatidic Acid. PLoS One. 2012 Oct 15;7(10).

90. Wang Y-C, Cui X-B, Chuang Y-H, Chen S-Y. Janus Kinase 3, a Novel Regulator for Smooth Muscle Proliferation and Vascular Remodeling. Arterioscler Thromb Vasc Biol [Internet]. 2017 Jul 1;37(7):1352–60. Available from: https://www.ahajournals.org/doi/abs/10.1161/ATVBAHA.116.308895

91. Kaplan DR, Morrison DK, Wong G, McCormick F, Williams LT. PDGF β-receptor stimulates tyrosine phosphorylation of GAP and association of GAP with a signaling complex. Cell. 1990 Apr 6;61(1):125–33.

92. Tallquist MD, French WJ, Soriano P. Additive Effects of PDGF Receptor β Signaling Pathways in Vascular Smooth Muscle Cell Development. PLOS Biol [Internet]. 2003;1(2):e52. Available from: https://journals.plos.org/plosbiology/article?id=10.1371/journal.pbio.0000052

93. Kim E, Goraksha-Hicks P, Li L, Neufeld TP, Guan K-L. Regulation of TORC1 by Rag GTPases in nutrient response. Nat Cell Biol 2008 108 [Internet]. 2008 Jul 6;10(8):935–45. Available from: https://www.nature.com/articles/ncb1753

94. Numaguchi K, Eguchi S, Yamakawa T, Motley ED, Inagami T. Mechanotransduction of rat aortic vascular smooth muscle cells requires RhoA and intact actin filaments. Circ Res [Internet]. 1999 Jul 9;85(1):5–11. Available from: http://www.circresaha.org

95. Qi YX, Jiang J, Jiang XH, Wang XD, Ji SY, Han Y, et al. PDGF-BB and TGF-β1 on cross-talk between endothelial and smooth muscle cells in vascular remodeling induced by low shear stress. Proc Natl Acad Sci U S A. 2011 Feb 1;108(5):1908–13.

96. Tang Y, Yang X, Friesel RE, Vary CPH, Liaw L. Mechanisms of TGF-β-Induced Differentiation in Human Vascular Smooth Muscle Cells. J Vasc Res [Internet]. 2011 Oct;48(6):485. Available from: /pmc/articles/PMC3169366/

97. Mao X, Debenedittis P, Sun Y, Chen J, Yuan K, Jiao K, et al. Vascular smooth muscle cell smad4 gene is important for mouse vascular development. Arterioscler Thromb Vasc Biol. 2012 Sep;32(9):2171–7.

98. Huang Y, Shen Z, Chen Q, Huang P, Zhang H, Du S, et al. Endogenous sulfur dioxide alleviates collagen remodeling via inhibiting TGF-β/Smad pathway in vascular smooth muscle cells. Sci Reports 2016 61 [Internet]. 2016 Jan 14;6(1):1–14. Available from: https://www.nature.com/articles/srep19503

99. Lu P, Wang S, Cai W, Sheng J. Role of TGF-β 1/Smad3 signaling pathway in secretion of type i and III collagen by vascular smooth muscle cells of rats undergoing balloon injury. J Biomed Biotechnol. 2012;2012.

100. Gong J, Zhou D, Jiang L, Qiu P, Milewicz DM, Chen YE, et al. In Vitro Lineage-Specific Differentiation of Vascular Smooth Muscle Cells in Response to SMAD3 Deficiency: Implications for SMAD3-Related Thoracic Aortic Aneurysm. Arterioscler Thromb Vasc Biol [Internet]. 2020;40(7):1651–63. Available from: https://pubmed.ncbi.nlm.nih.gov/32404006/

101. Croker BA, Kiu H, Nicholson SE. SOCS Regulation of the JAK/STAT Signalling Pathway. Semin Cell Dev Biol [Internet]. 2008;19(4):414. Available from: /pmc/articles/PMC2597703/

102. Feinberg MW, Watanabe M, Lebedeva MA, Depina AS, Hanai J, Mammoto T, et al. Transforming Growth Factor-β1 Inhibition of Vascular Smooth Muscle Cell Activation Is Mediated via Smad3 *. J Biol Chem [Internet]. 2004 Apr 16;279(16):16388–93. Available from: http://www.jbc.org/article/S0021925820883641/fulltext

103. Thien A, Prentzell MT, Holzwarth B, Kläsener K, Kuper I, Boehlke C, et al. TSC1 Activates TGF-β-Smad2/3 Signaling in Growth Arrest and Epithelial-to-Mesenchymal Transition. Dev Cell. 2015 Mar 9;32(5):617–30.

104. Seay U, Sedding D, Krick S, Hecker M, Seeger W, Eickelberg O. Transforming Growth Factor-β-Dependent Growth Inhibition in Primary Vascular Smooth Muscle Cells Is p38-Dependent. J Pharmacol Exp Ther [Internet]. 2005 Dec 1;315(3):1005–12. Available from: https://jpet.aspetjournals.org/content/315/3/1005

105. Díez RR, Rodrigues-Díez R, Lavoz C, Rayego-Mateos S, Civantos E, Rodríguez-Vita J, et al. Statins Inhibit Angiotensin II/Smad Pathway and Related Vascular Fibrosis, by a TGF-β-Independent Process. PLoS One [Internet]. 2010;5(11):e14145. Available from: https://journals.plos.org/plosone/article?id=10.1371/journal.pone.0014145
